# Gegen Qinlian standard decoction alleviated irinotecan-induced diarrhea via PI3K/AKT/NF-κB axis by network pharmacology prediction and experimental validation combination

**DOI:** 10.1186/s13020-023-00747-3

**Published:** 2023-04-27

**Authors:** Jiamei Chen, Min Li, Rong Chen, Ziyi Xu, Xiaoqin Yang, Huan Gu, Lele Zhang, Chaomei Fu, Jinming Zhang, Yihan Wu

**Affiliations:** 1 State Key Laboratory of Southwestern Chinese Medicine Resources, Pharmacy School, Chengdu University of Traditional Chinese Medicine, No. 1166 Liutai Avenue, Wenjiang District, Chengdu, 611137 China; 2 School of Medicine, Chengdu University, Chengdu, 610106 China

**Keywords:** Gegen Qinlian standard decoction (GQD standard decoction), Active components, CPT-11-induced diarrhea, Network pharmacology, Mechanisms of action

## Abstract

**Background:**

The frequently occurred chemotherapy-induced diarrhea (CID) caused by irinotecan (CPT-11) administration has been the most representative side-effects of CPT-11, resulting in the chemotherapy suspension or failure. Our previous studies indicated that Gegen Qinlian formula exhibited a significant alleviation effect on CPT-11-induced diarrhea. However, referencing to Japanese Kampo medicine, the TCM standard decoction would supply the gap between ancient preparation application and modern industrial production.

**Methods:**

The LC–MS technology combined with network pharmacology was employed to identify the active ingredients and mechanisms of GQD standard decoction for CPT-11-induced diarrhea. The anti-inflammatory activities associated with intestinal barrier function of GQD standard decoction were studied by SN-38 activated NCM460 cells in vitro and CPT-11-induced diarrhea in vivo. Proteins involved in inflammation, mRNA levels, disease severity scores, and histology involved in intestinal inflammation were analysed.

**Results:**

There were 37 active compounds were identified in GQD standard decoction. Network pharmacology analyses indicated that PI3K-AKT signaling pathway were probably the main pathway of GQD standard decoction in CPT-11-induced diarrhea treatment, and PIK3R1, AKT1, NF-κB1 were the core proteins. Moreover, we found that the key proteins and pathway predicted above was verified in vivo and in vitro experiments, and the GQD standard decoction could protect the cellular proliferation in vitro and ameliorate CPT-11-induced diarrhea in mice model.

**Conclusions:**

This study demonstrated the molecular mechanism of 37 active ingredients in GQD standard decoction against CPT-11-induced diarrhea. And the core proteins and pathway were validated by experiment. This data establishes the groundwork for particular molecular mechanism of GQD standard decoction active components, and this research can provide a scientific reference for the TCM therapy of CID.

**Supplementary Information:**

The online version contains supplementary material available at 10.1186/s13020-023-00747-3.

## Introduction

Chemotherapy-induced diarrhea (CID), a well-recognized common side effect of most chemotherapeutic regimens [1], significantly delay duration of therapy and decrease patient compliance, thereby interfering with the complete course of treatment and ultimately increasing the cost of care [2]. Particularly, the delayed diarrhea is frequently occurred in patients administrated with CPT-11, as a first-line therapeutic agent for colorectal cancer [3, 4]. It has been reported that approximately 87% colorectal cancer patients receiving CPT-11 were suffered severe diarrhea (grade 3/4), due to the accumulation of its active metabolite SN-38 in gut, resulting in the intestinal mucosal damage. Despite some strategies and agents have been attempted to treat CPT-11-induced delayed diarrhea, such as loperamide, atropine and specific antidiarrheal agents [5], their therapeutic outcomes are still greatly limited [5], as well as these caused side-effects. Therefore, no standard therapy has been generally adopted.

At present, traditional herbal medicine has received considerable attention in relieving the side-effects of chemotherapy [6]. Extensive studies have shown that several TCM herbal formulas such as Banxia Xiexin decoction [7], Huangqin decoction [8], Shengjiang Xiexin decoction [9], etc., exhibited the significant effects to reduce CPT-11-induced diarrhea. The most successful application case would be that Prof. Yung-Chi Cheng’s team have demonstrated the alleviation effects of PHY-906, an extract of Huangqin decoction, on gastrointestinal toxicity induced by CPT-11 from bench to bedside [10, 11], and even developing a new drug YIV-906 approved by FDA. In our previous studies, we have revealed that the similar clinical value of a TCM formulation Gegen Qinlian standard decoction (GQD standard decoction), which was recorded in the same classics (“*Treatise on Febrile Diseases*”) with Huangqin decoction in Han Dynasty (202 BC-220 AD) [12]. Both the microporous resin extract fraction and the pill preparation of Gegen Qinlian could alleviate CPT-11-induced diarrhea in mice [13], involving in regulating inflammation, oxidative stress, and proliferation processes, activating Keap1/Nrf2 pathway, up-regulating the intestinal barrier function, and inhibiting hCE2 activity [12]. CID is often accompanied with enteritis, the evidences PI3K/Akt/NF-κB signal pathway participate in the pathogenesis of diarrhea-related diseases by regulating various inflammatory factors. Cytokines, viruses, and other bio-factors can lead to phosphorylation of PI3K and AKT [14], which can activate NF-κB, and the PI3K/Akt/NF-κB signal pathway can become a target for treatment of CID or screening drugs. It is unknown whether GQD standard decoction can alleviate CID via PI3K/Akt/NF-kB signal pathway [15]. However, the clinical application of Gegen Qinlian standard decoction for CPT-11-induced diarrhea treatment is still challenging. On the one hand, currently the microporous resin extract of Gegen Qinlian can only be utilized in pre-clinical study, because of the lack of standardized production. On the other hand, Gegen Qinlian Pill, a commercial modern preparation, confronts the different extraction approach from the corresponding record in “*Treatise on Febrile Diseases*”, resulting in the different chemical constituents.

Commonly, decoction is one of the most widely used TCM dosage forms in clinics. Nevertheless, the different preparation conditions such as herbal dosage, decocting time and solvent, are apt to result in the varied decoction quality as well as the therapeutic outcomes [16]. To maintain the consistency on therapeutic effectiveness between the decoction sample based on ancient record and that derived from modern industrial preparation, the concept of “standard decoction” has been broadly in Japanese Kampo medicine [17]. Meanwhile, to research and develop the ancient TCM classical prescriptions, based on “standard decoction” pattern, has currently become a most important field in new drug development. Therefore, to prepare the standard decoction based on the ancient decoction preparation condition and investigate its mitigation effects on CPT-11-induced diarrhea as well as the potential mechanisms would be meaningful for the following clinical application of GQD standard decoction.

**Fig. 1:**
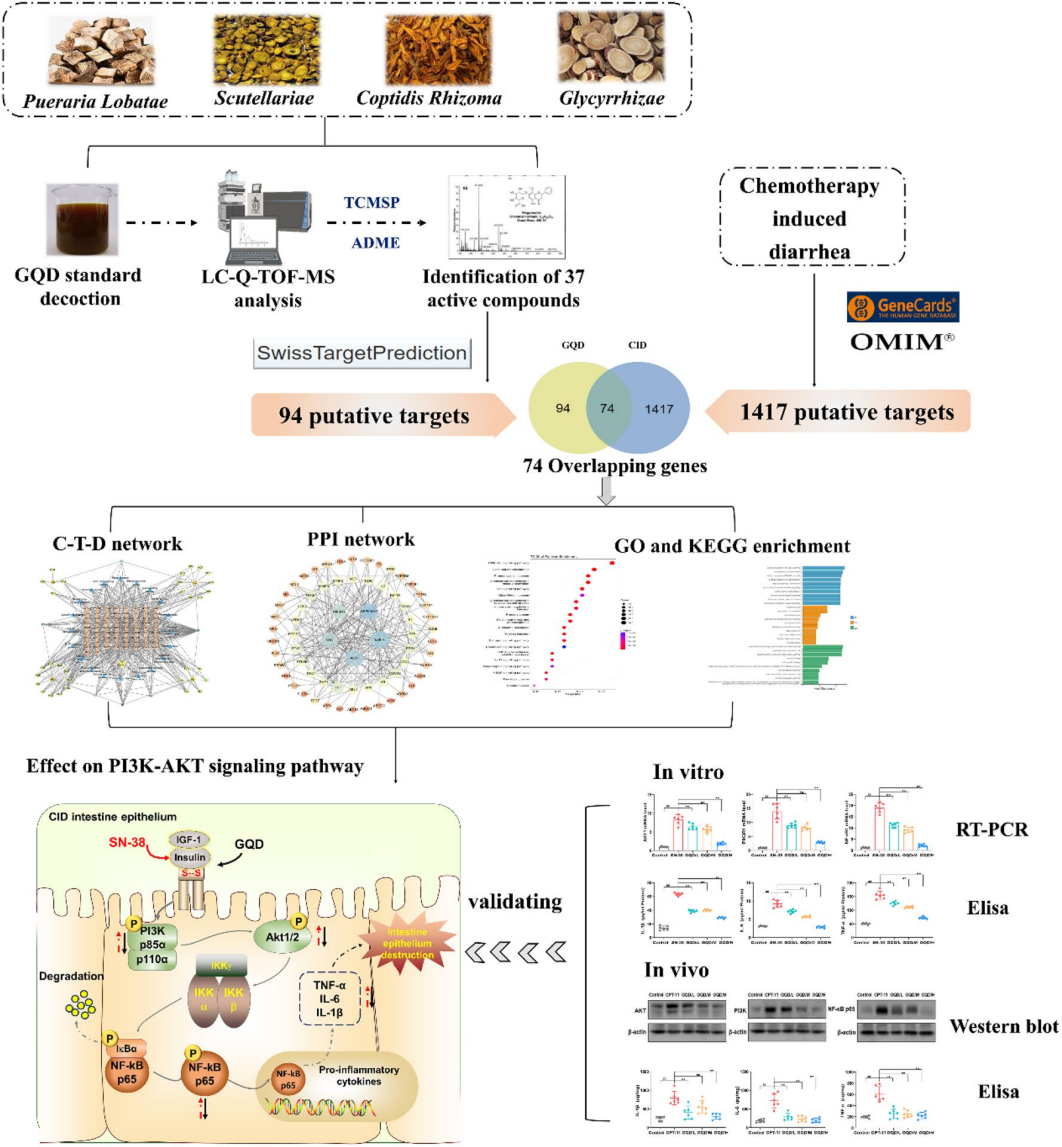
Exploration of the amelioration effects and potential mechanisms of GQD standard decoction on CPT-11 induced diarrhea, by combination of network pharmacology prediction and experimental validation

As well-known, the mechanisms of TCM would be complex, due to the multiple chemical components, and the related multiple proteins and signal pathways. Using traditional biological experimental methods to investigate the mechanism of TCM possess the limitation [18–20]. To explore the mechanisms of GQD standard decoction based on the combination of network pharmacology prediction and the experimental verification would provide a useful approach. As proof of concept, herein we revealed the mechanisms of GQD standard decoction for the prevention of CPT-11-induced diarrhea. The design illustration was shown in Fig. 1. Primarily, GQD standard decoction was prepared according to the record in “*Treatise on Febrile Diseases*”, based on our previous textual research. And then, LC–MS technology was employed to identify the chemical constituent profile in. Based on it, the compounds-targets-diseases(C-T-D) network was constructed [21], in which the key proteins and signaling pathways were presented, providing the predicted pathways. Most importantly, we found that the prepared GQD standard decoction could protect the cellular proliferation in vitro and ameliorate CPT-11-induced diarrhea in mice model, in which the key proteins and pathway predicted above was verified.

## Materials and methods

### Chemicals and reagents

CPT-11 (Irinotecan HCl Trihydrate, *M*_w_ 677.19) was purchased from Melonepharma Co., Ltd. (Dalian, China). Weikeqi Biological Technology Co., Ltd (Chengdu, China) supplied the reference compounds Puerarin, Coptisine, Berberine, Palmatine, Daidzin, Liquiritin, Daidzein, Wogonoside, Wogonin, Baicalin and Baicalein (purity ≥ 98%). HPLC-grade reagent were obtained from Thermo Fisher Scientific (Thermo scientific, USA). The tumor Necrosis factor-α (TNF-α) kit, interleukin 1β (IL-1β) kit, and interleukin 6 (IL-6) kit were purchased from Multi Science (Lianke) Biotech Co., Ltd. (Hangzhou, China). PI3K antibody (60225-1-Ig), NF-κB p65 antibody (66535-1-Ig) were purchased from Proteintech Group, Inc (Wuhan, China). Abmart Shanghai Co., Ltd (Shanghai, China) provided the AKT (T55561) antibody and Servicebio Biotechnology Co., Ltd. (Wuhan, China) provided the β-actin antibody (GB12001). Professor Guihua Jiang (College of Pharmacy, Chengdu University of Traditional Chinese Medicine, China) identified all of the herbs procured from the Sichuan Neautus Traditional Chinese Medicine Co., Ltd. (Chengdu, China).

### Cells and animals

The Human normal colonic epithelial cell (NCM 460) was purchased from Wuhan Fine Biological Technology Co., Ltd. (Wuhan, China). RPMI-1640 complete medium containing 10% heat-inactivated fetal bovine serum (FBS) and 1% penicillin–streptomycin was used to cultivate NCM 460 cells. The cells were kept at 37 °C in an environment that was humidified and added to 5% CO2.

Male ICR mice (weight 22 ± 2 g) were purchased from SPF (Beijing) Biotechnology Co., Ltd. (Beijing, China). (quality certification number: SCXK 2019-0010). The mice were housed in cages at 22 ± 2 ℃, 55% ± 5% relative humidity and with a 12 h light–dark cycle. The Institutional Animal Care and Use Committee (IACUC) of Chengdu University of Traditional Chinese Medicine provided the guidelines for all in vivo tests.

### Preparation of GQD standard decoction

The GQD standard decoction extract was performed in accordance with ancient records [22]. The herbal material Pueraria lobata (Willd.) Ohwi (Ge-Gen, GG, 110.4 g) was firstly soaked in distilled water (1.6 L) for 30 min and then boiled to a solution volume of 1.2 L. Next, the other herbs, including *Coptis chinensis* Franch. (Huang-Lian HL, 41.4 g), *Scutellaria baicalensis* Georgi (Huang-Qin, HQ, 41.4 g) and *Glycyrrhiza uralensis* Fisch. (Gancao, GC, 27.6 g) were added in solution at the weight ratio of 8:3:3:2 and boiled for 3 h, When the volume of decoction is boiled to 0.4 L, the aqueous extract was filtered by gauze, then lyophilized. Ultrasonic extraction was used to dissolve 0.1 g of lyophilized GQD standard decoction powder in 10 mL of 70% methanol. The supernatant was filtered, and analyzed by LC–MS.

### UPLC-Q-TOF–MS analysis

The Vanquish UHPLC system (Thermo Fisher Scientific) couppled with Q Exactive quadrupole-electrostatic field orbitrap mass spectrometer was used to evaluate GQD standard decoction powder. Samples were performed on a SunFire C18 column (3.0 × 150 mm, 3.5 μm, Waters, Massachusetts, USA). The column temperature was 30 ℃ and flow rate was 0.3 mL/min. The mobile phases A and B were deionized water with 0. 1% formic acid and acetonitrile with 0.1% formic acid, respectively. Installing the gradient elution program: 0–3 min, 10%–12% B; 3–5 min, 12%–13% B; 5–8 min, 13%–16% B; 8–16 min, 16%–20% B; 16–18 min, 20%–21% B; 18–20 min, 21% B; 20–38 min, 21%–40% B; 38–50 min, 40%–50% B; 50–54 min, 50%–51% B; 54–60 min, 51%–90% B.

This MS system operated in positive ionization modes with m/z 100–1500. Positive and negative ion spray modes were 3.2 and 2.5 kV. 350 °C for the ion source heating. Fragment voltage was 50 V, and the scan mass ratio was within m/z 50–1500.

The mass detection was carried out with a m/z tolerance of 10 ppm and an RT tolerance of 0.01 min, and the noise level set to 1.5. Finally, the mgf data file and its associated. csv metadata file, which included Peaks height and areas integration, were exported.

### Quantification 11 ingredients identified with the reference standard of GQD standard decoction

#### HPLC instrumentation and conditions

The HPLC determination conditions were exhibited in Additional file 1: Material S1.

#### Methodological investigation

According to the recommendations of Chinese Pharmacopoeia, the specificity, linearity, instrument precision, method repeatability, sample stability and method recovery of the high performance liquid phase content determination method were verified.

#### Content determination of 11 components

The above method was used to prepare 15 batches of sample solutions of 11 ingredients identified with the reference standard in GQD standard decoction. Among them, 10 μL was injected under high performance liquid phase conditions, the peak area of each compound was measured, and the content of 11 ingredients of multiple batches of GQD standard decoction was calculated.

### Network pharmacology analysis

#### Screening the components of GQD standard decoction

The GQD standard decoction’s candidate compounds were recognized by LC–MS. And the TCMSP (https://old.tcmsp-e.com/tcmsp.php) was used to obtain compounds' pharmacological information. For the efficacy prediction, based on the body’s drugs ADME, compounds with OB ≥ 30% and DL ≥ 0.18 were chosen. And the active compounds information was queried and standardized by the Pubchem database (https://pubchem.ncbi.nlm.nih.gov/).

#### Prediction of potential proteins

The proteins of active component was obtained from SWISS Target Prediction database (http://swisstargetprediction.ch/), and collected the top 15 proteins for docking scores or the proteins with a docking score of 1. The related disease proteins were searched from two databases using “chemotherapy induced diarrhea” as the keywords: the GeneCards database (http://www.genecards.org) OMIM database (http://www.omim.org). The GQD standard decoction absorbable component proteins and CID-related proteins were intersected to yield possible GQD treatments CID proteins, which were then displayed in VENNY2.1.

#### GO and KEGG enrichment analysis

The clusterProfiler R package was used to evaluate the biological consequences of GQD standard decoction for CID treatment. And GO and KEGG pathways with P < 0.05 was identified.

#### Construction of compounds-targets-diseases network

Cytoscape 3.7.2 showed the following network construction. The PPI of GQD standard decoction in treating CID were constructed by STRING database (https://string-db.org) and Cytoscape, in which the network proteins were screened with a combined score > 0.7. The compounds-targets-diseases (C-T-D) network was visualized with Cytoscape software and analyzed with Network Analyzer.

### Cell viability analysis

NCM 460 cells (6000 cells/well) were planted in 96-well plates overnight for adherence. After 12 h, cells were cultured with a fresh culture medium, SN-38, GQD standard decoction @SN-38. After 24 h of treatment, CCK8 (Beyotime, Shanghai, China) was given to cells for 4 h at 37 °C, and the optical density was measured at 450 nm with a microplate plate reader (Thermo Fisher Scientific, Inc.).

### Prevention effects of GQD standard decoction on NCM460 cell damage

NCM 460 cells were planted in 24-well plates (2 × 10^5^ cells/well). Cells were cultured with a fresh culture medium, SN-38, GQD standard decoction @SN-38 (500 nmol/L) after overnight culture. The equivalent amounts of GQD standard decoction in various groups were 18.75, 37.5 and 75 µg/ml. Untreated cells were utilized as control. After 24 h, each well’s cell supernatant was collected. ELISA kits were used to quantify TNF-α, IL-1β, IL-6 in the cell supernatants.

### Real-time PCR analysis

Animal Total RNA Isolation Kit (Foregene, Chengdu, China) was used to extract and purify Total RNA from NCM 460 cells. The 5*All-In-One MasterMix (abm, USA) was used to synthesize complementary DNA (cDNA) Then, water that was devoid of nucleases diluted cDNA. Real-time PCR was performed on an Applied Biosystems 7500 machine (Life Technologies) using 2 × SYBR Green qPCR Master Mix (Servicebio, Wuhan, China). Each gene’s e relative mRNA expression was determined in relation to β-Actin mRNA, and all genes are shown as fold changes from the negative control group. Each sample's mean was determined by six replicate experiments. The results are expressed using the relative quantitative analysis of 2(− ΔΔCT). All PCR primers are summarized in Table 1.

**Table 1:** Primers and probes for real-time PCR

Gene	Primer sequence (5′–3′)	GenBank accession no	Product size (bp)
AKT1	AGCGACGTGGCTATTGTGAAG GCCATCATTCTTGAGGAGGAAGT	NM_001014431	96
PIK3R1	TGGACGGCGAAGTAAAGCATT AGTGTGACATTGAGGGAGTCG	NM_181523	154
NF-κB1	ATGTGGAGATCATTGAGCAGC CCTGGTCCTGTGTAGCCATT	NM_001145138	151
β-Actin	AATCTGGCACCACACCTTCTACAA GGATAGCACAGCCTGGATAGCAA	NM_031144	172

### Effects on CPT-11-induced diarrheal mice model

After one week of adaptation, the mice were divided into five groups at random (n = 5): Control, CPT-11, GQD-L (0.7 g/kg), GQD-M (1.4 g/kg), GQD-H (2.8 g/kg). The CPT-11 was dissolved in water plus 1% Tween and the GQD standard decoction powder were dissolved in water. In the control group, the mice were only given water plus 1% Tween (10 ml/kg, i.p.) from days 7 to 10 and water (10 ml/kg, i.g) from days 1 to 14. In the CPT-11 and GQD standard decoction-treated groups, the mice received injection of CPT-11 suspensions (50 mg/kg, i.p.) from days 7 to 10, and GQD standard decoction extract were intra-gastric administration to mice in GQD standard decoction-treated groups from days 1 to 14. The GQD standard decoction’s dose was based on preliminary experiment and the group’s previous study [13]. Body weight, stool consistency, and fecal hemorrhage were monitored daily throughout the treatment period. The disease activity index (DAI) provides a complete evaluation of body weight loss (0−4), stool consistency state (0−4), and fecal bleeding (0−4) [23]. At day 15, all mice were sacrificed by euthanasia with isoflurane, then, the duodenum and colon were collected.

### Histopathological analysis

Each group's colon tissues were photographed and sized. And 1.0 cm samples of duodenum and colon will be collected and preserved in 4% paraformaldehyde for 24 h and embedded in paraffin. And samples will be processed for hematoxylin–eosin (H&E) staining for general morphological observations. And mucin content was quantified using Periodic acid–Schiff staining (PAS) staining.

### ELISA determination

Weighing and homogenizing the distal colon tissues in PBS. After centrifugation at 3000 rpm for 30 min, then, supernatant was collected for IL-6, TNF-α, IL-1β concentration measurement.

### Immunohistochemical analysis

Immunohistochemical staining of paraffin slices revealed Occludin, ZO-1. Paraffin sections were blocked in 10% normal rabbit serum + 5%nonfat dry milk + 3% BSA + 0.1% Triton X-100 for 30 min, and then treated overnight with anti-Occludin, ZO-1 at 4 ℃. The slices were rinsed three times with PBS and HRP-goat anti-rabbit IgG antibody (1:200) was incubated for 50 min. The sections were treated with diamino-benzidine and counterstained with hematoxylin for 3 min, and visualized using the Nikon DS-U3 microscope system (Tokyo, Japan).

### Western blot assays

Colon tissue was extracted with RIPA buffer with protease inhibitor. After high-speed centrifugation, BCA protein assay kit (Biosharp, China) was use to determine protein content. Protein suspension (30 µg) combined with 5× loading buffer (Biosharp, China) was electrophoresed on sodium dodecyl sulfate-polyacrylamidegel (SDS-PAGE, 7.5–10%) and transferred to 0.22 µm PVDF membranes (Millipore, USA). Membranes were occluded with 5% nonfat milk for 2 h and kept at 4 °C overnight. After washing with TBST (0.1% Tween), the membrane was incubated with HRP-conjugated secondary antibodies for 1 h at room temperature. The ChemiDocTM XRS + system (Bio-Rad) was used to observe immunoreactive bands with HRP substrate (Luminata, Millipore). ImageJ 1.48 (National Institutes of Health, Bethesda, MD, USA) software examined protein expression levels. The results are representative Mouse anti-PI3K (1:5000), rabbit anti-AKT (1:1000), Mouse anti-NF-κB p65 (1:2000), and three independent experiments used β-Actin levels as controls.

### Statistical analysis

All data were expressed as mean ± SEM. Comparing several groups with one-way analysis of variance (ANOVA). P < 0.05 was considered statistically significant. Statistical analysis and graphing were performed using GraphPad Prism 8.0 software (San Diego, CA, USA).

## Results

### Identification compounds of GQD standard decoction by UPLC-Q-TOF/MS

**Fig. 2:**
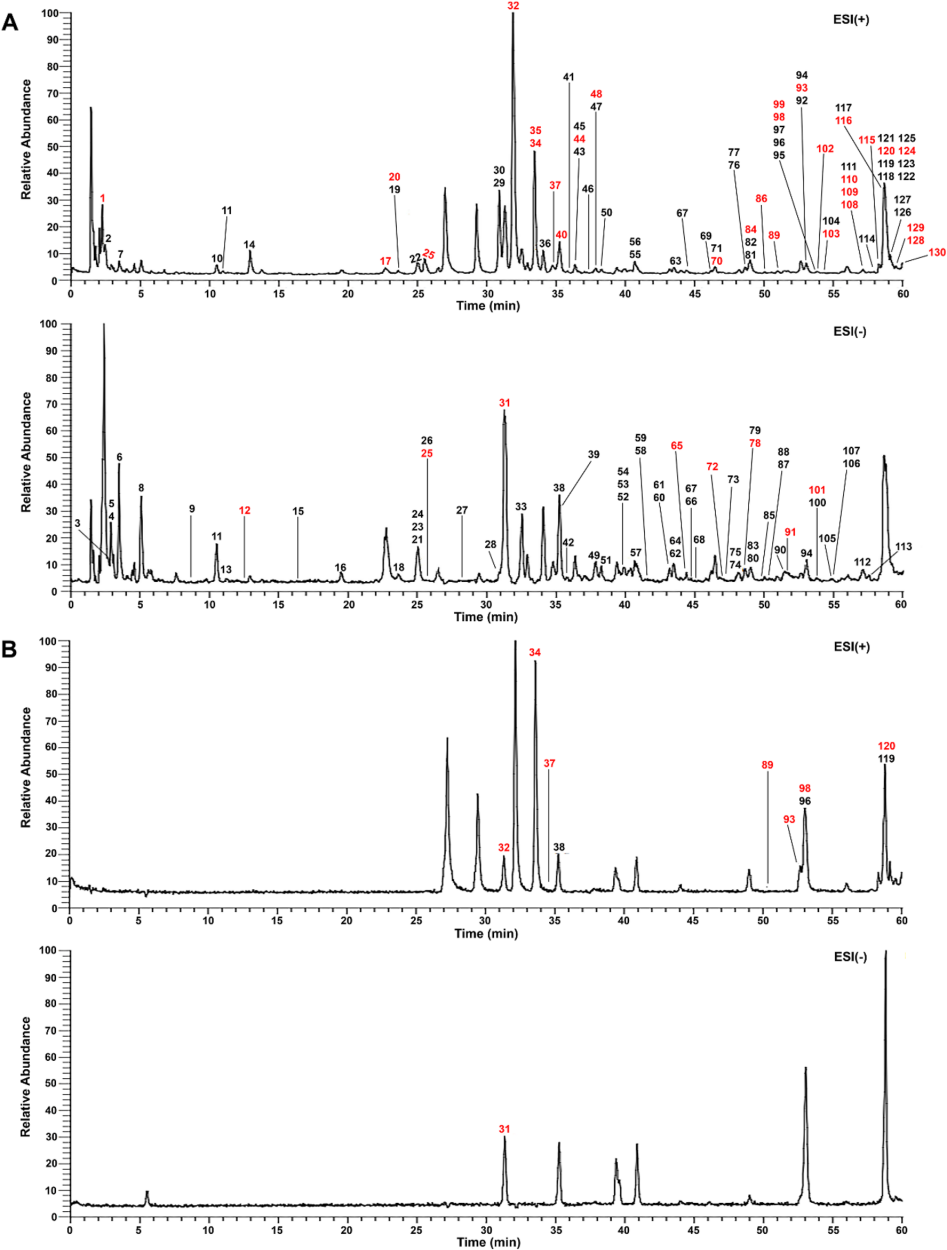
**A** Total ion chromatographs of GQD standard decoction in positive mode and negative mode. **B** 11 mixed standards including Puerarin, Coptisine, Berberine, Palmatine, Daidzin, Liquiritin, Daidzein, Wogonoside, Wogonin, Baicalin, Baicalein, based on UPLC-Q-TOF/MS chromatogram in positive ion mode and in negative ions mode. Red numbers represent the potential bioactive components

UPLC-Q-TOF/MS representative chromatograms are shown in Fig. 2. Based on literature, database matching, and reference standards, 130 components were found in GQD standard decoction. Additional file 1: Table S1 lists compounds identified from GQD standard decoction, including 75 flavonoids, 13 alkaloids, 10 carboxylic acids, 5 glycosides, 4 coumarins, 2 phenylethanol glycosides, 1 phenylpropanoid, 1 saponins and other types of compounds. And 11 chemicals were discovered by comparing retention times with reference standards (Additional file 2: Fig. S1). In the 130 identified compounds, the number of chemical constituents of HQ is 44, GC is 39, GG is 35, HL is 19, which suggested that the main sources of detected chemicals are HQ, GC, GG.

### Quantitative analysis of 11 ingredients identified with the reference standard

For further quantification 11 ingredients identified with the reference standard, the quantification of 11 ingredients of multiple batches of GQD standard decoction was established. At the same time, through the optimization of high performance liquid phase conditions, the chromatographic peaks of these 11 components have good resolution and detection performance. All the reference substances had a good linear relationship within the measurable range (r > 0.9997), which verified the effectiveness of the method. In addition, this measurable method has good specificity, linearity, instrument precision, method repeatability, sample stability and method recovery. Meanwhile, the 11 components in GQD standard decoction were quantified by the above method. The contents of 11 chemicals in GQD standard decoction in 15 batches of GQD standard decoction were listed in Additional file 1: Table S2.

### Network pharmacology analysis

#### Identification of active compounds proteins of GQD standard decoction in CPT-11-induced diarrhea conditions

**Fig. 3:**
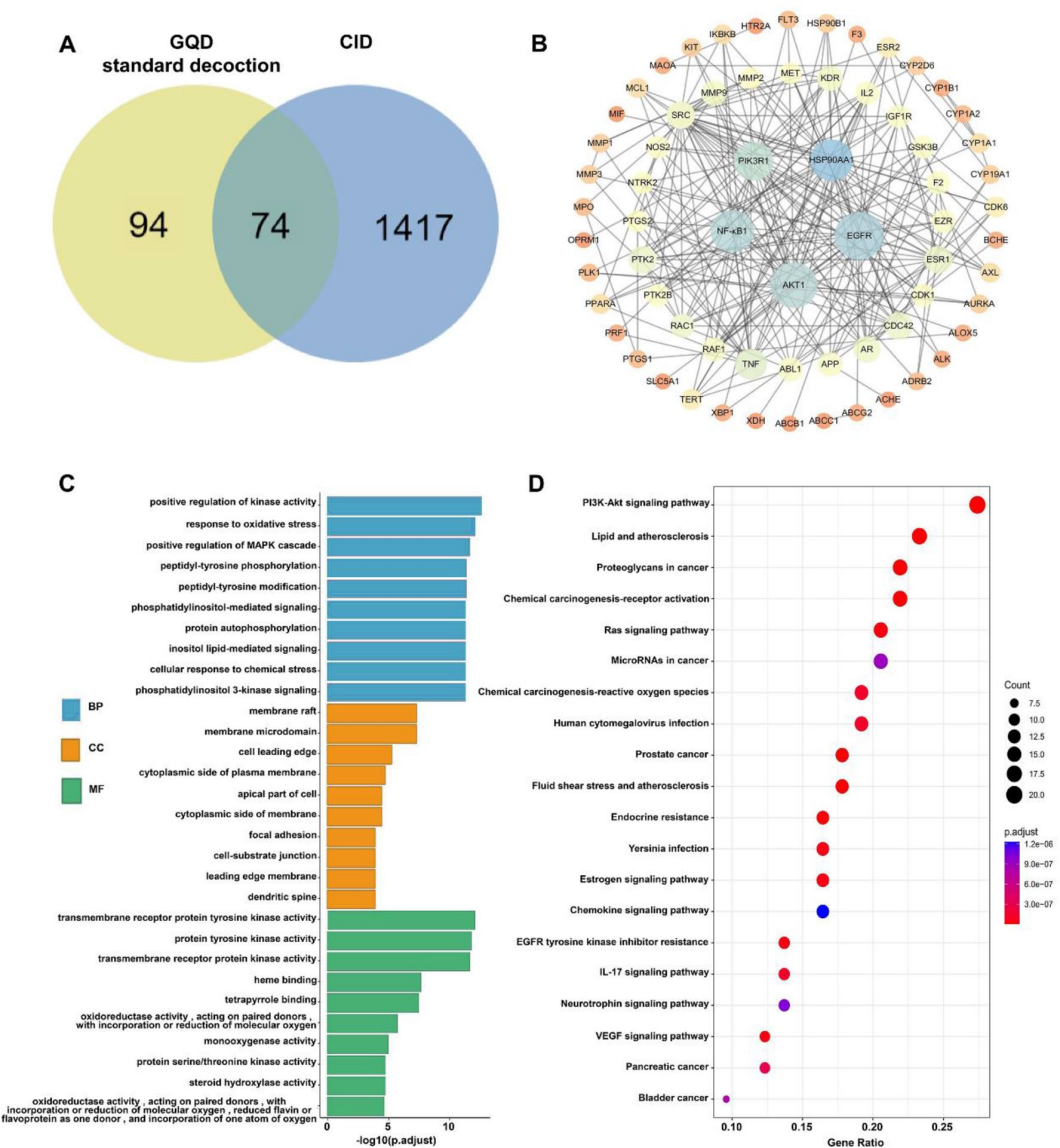
Network pharmacology prediction for GQD standard decoction treatment of CID. **A** Venn analysis showing the crosstalk between GQD standard decoction and CID. **B** The PPI network of proteins downloaded from STRING database and imported into Cytoscape 3.7.2. In descending degree order, node colors are blue, yellow, and orange. **C** The top 10 enriched GO terms of potential proteins of GQD standard decoction in BP, CC, and MF of CID. **D** The top 20 pathways related to the effect of GQD standard decoction against CID

Among 130 constituents, 37 compounds met the requirements of OB ≥ 30% and DL ≥ 0.18. These components were selected for further exploration (Table 2). Furthermore, the proteins of 37 active compounds in GQD standard decoction were gathered from SwissTarget Prediction and TCMSP databases, 94 predictive proteins were identified (Additional file 1: Table S3). Additionally, we collected 283 CID-associated proteins from the OMIM database and 1492 CID-associated proteins genes from GeneCards. 1417 disease protein genes were obtained after duplicates were eliminated (Additional file 3: Material S1). A Venn diagram was made using the 1417 disease proteins and the 94 active compound proteins (Fig. 3A). A total of 74 overlapping proteins were obtained, and these were chosen as the main genes for further study (Additional file 1: Table S4).

**Table 2:** UPLC-Q-TOF/MS was used to identify the 37 potential bioactive components in GQD standard decoction

No	t_R_ (min)	Compound	Molecular formula	[M + H]^+^/ [M-H]^−^	Fragment ions (m/z)	Source
1	2.31	LicochalconeA	C_21_H_22_O_4_	339.1591/	339.1049	GC
12	12.01	LicoisoflavoneB	C_20_H_16_O_6_	/351.0877	153.0551	GC
17	22.69	Berlambine	C_20_H_17_NO_5_	352.1179/	337.0943,308.0918	HL
20	23.64	Daidzein-4',7-diglucoside	C_27_H_30_O_14_	579.1708/	417.1178,255.0650	GG
25	25.50	Berberrubine	C_19_H_16_NO_4_	323.1152/321.1006	307.0838,279.0889	HL
31	31.33	Puerarin	C_21_H_20_O_9_	/415.1034	267.0666,295.0614,277.0508	GG
32	31.88	Coptisine	C_19_H_14_NO_4_	321.0995/	292.0948,290.0812	HL,HQ
34	33.47	Berberine	C_20_H_18_NO_4_	337.1308/	321.0996,320.0917,292.0967	HL
35	33.47	Epiberberine	C_20_H_18_NO_4_	337.1308/	320.0917,292.0967	HL,HQ
37	34.56	Palmatine	C_21_H_22_NO_4_	353.1621/	337.0941,336.0710,308.1281	HL
40	35.45	Worenine	C_20_H_15_NO_4_	334.1073/	304.0603,302.0812,290.0811,261.0800	HL
44	36.37	3'-Methoxydaidzein	C_16_H_12_O_5_	285.0768/	270.0522	GG
48	37.85	Moslosooflavone	C_17_H_14_O_5_	299.0914/	315.0888,274.1656	HQ
65	44.39	Glycyroside	C_27_H_30_O_13_	/561.1613	267.0665,252.0430	GC
70	46.8	5,7,2',5-tetrahydroxy-8,6-dimethoxyflavone	C_17_H_14_O_8_	347.0761/	332.0527,314.0420	HQ
72	47.09	5,7,4'-trihydroxy-8-methoxyflavone	C_16_H_12_O_6_	/299.0561	284.0329	HQ
78	48.64	Formononetin	C_16_H_12_O_4_	/267.0662	252.0429,223.0400,195.0449	GG,GC
84	49.35	Moupinamide	C_18_H_19_NO_4_	314.1753/	314.1384	HL
86	50.35	Quercetin	C_15_H_10_O_7_	303.0499/	303.0848	GC,HL
89	50.96	Liquiritin	C_21_H_22_O_9_	418.1258/	257.0808,163.0385,137.0234	GC
91	51.83	LicochalconeB	C_16_H_14_O_5_	/285.0765	150.0316,191.0352	GC
93	53.06	Daidzein	C_15_H_10_O_4_	255.0651/	255.0653,157.0647,137.0235	GG
98	53.85	Wogonin	C_16_H_12_O_5_	285.0763/	285.0758	HQ
99	53.85	OroxylinA	C_16_H_12_O_5_	285.0760/	270.0524	HQ
101	53.88	Calycosin	C_16_H_12_O_5_	/283.0613	268.038	GC,HQ
102	53.88	Acacetin	C_16_H_12_O_5_	283.0611/	268.038	HQ,HL
103	54.25	Panicolin	C_17_H_14_O_6_	315.0863/	300.0631,285.0391	HQ
108	57.12	Dihydrobaicalin	C_21_H_20_O_11_	449.1078/	273.0758	HQ
109	57.14	Naringenin	C_15_H_12_O_5_	273.0757/	131.0493	GC
110	57.14	Carthamidin	C_15_H_12_O_6_	289.0707/	271.0603	HQ
115	58.31	Glycyrol	C_21_H_18_O_6_	367.1176/	339.1234	GC
116	58.6	Lupiwighteone	C_20_H_18_O_5_	339.1227/	147.0900,119.0827	GC
120	58.67	Baicalein	C_15_H_10_O_5_	271.0600/	253.0499	HQ
124	58.67	Norwogonin	C_15_H_10_O_5_	271.0600/	253.0499,169.0132,123.0079	HQ
128	59.64	DihydrooroxylinA	C_16_H_14_O_5_	287.0914/	183.0288,131.0492,103.0546	HQ
129	59.77	SemilicoisoflavoneB	C_20_H_16_O_6_	353.1019/	257.5884	GC
130	59.95	GancaoninA	C_21_H_20_O_5_	353.1383/	337.1263,297.2234	GC

#### Characterization of potential therapeutic proteins of GQD standard decoction

To fully understand the potential mechanism by which GQD standard decoction treats CPT-11-induced diarrhea, the gene names of 74 GQD standard decoction anti-CID proteins were imported into the STRING database, and the minimum needed interaction score was set as 0.7. Using Cytoscape software 3.7.1 according to the protein degree, the PPI network was constructed (Fig. 3B). With this network, HSP90AA1, EGFR, AKT1, PIK3R1, NF-κB1 were identified as core proteins with degree value more than 7.1 (the mean of degree). In the GO enrichment analysis, including 1427 biological process (BP) terms; 64 cell component (CC) terms; 90 molecular function (MF) terms, and the top 10 highly enriched terms in the BP, CC and MF are shown (Fig. 3C). KEGG analysis enrichment analysis showed that 135 pathways (P < 0.05) were identified (Additional file 1: Table S5) and 20 pathways (Fig. 3D) were screened out (P < 0.05). According to KEGG study, the effect of GQD standard decoction was mostly immunological and diseases-related pathways, with diseases-related pathways primarily included cancer, infection, and inflammatory bowel disease. Immune-related pathways mainly included PI3K/AKT, EGFR signaling pathways. Interestingly, we found that PI3K/AKT signaling pathway is closely connected to the above signaling pathways [17, 18, 21].

#### Network construction of compound-target-disease

**Fig. 4:**
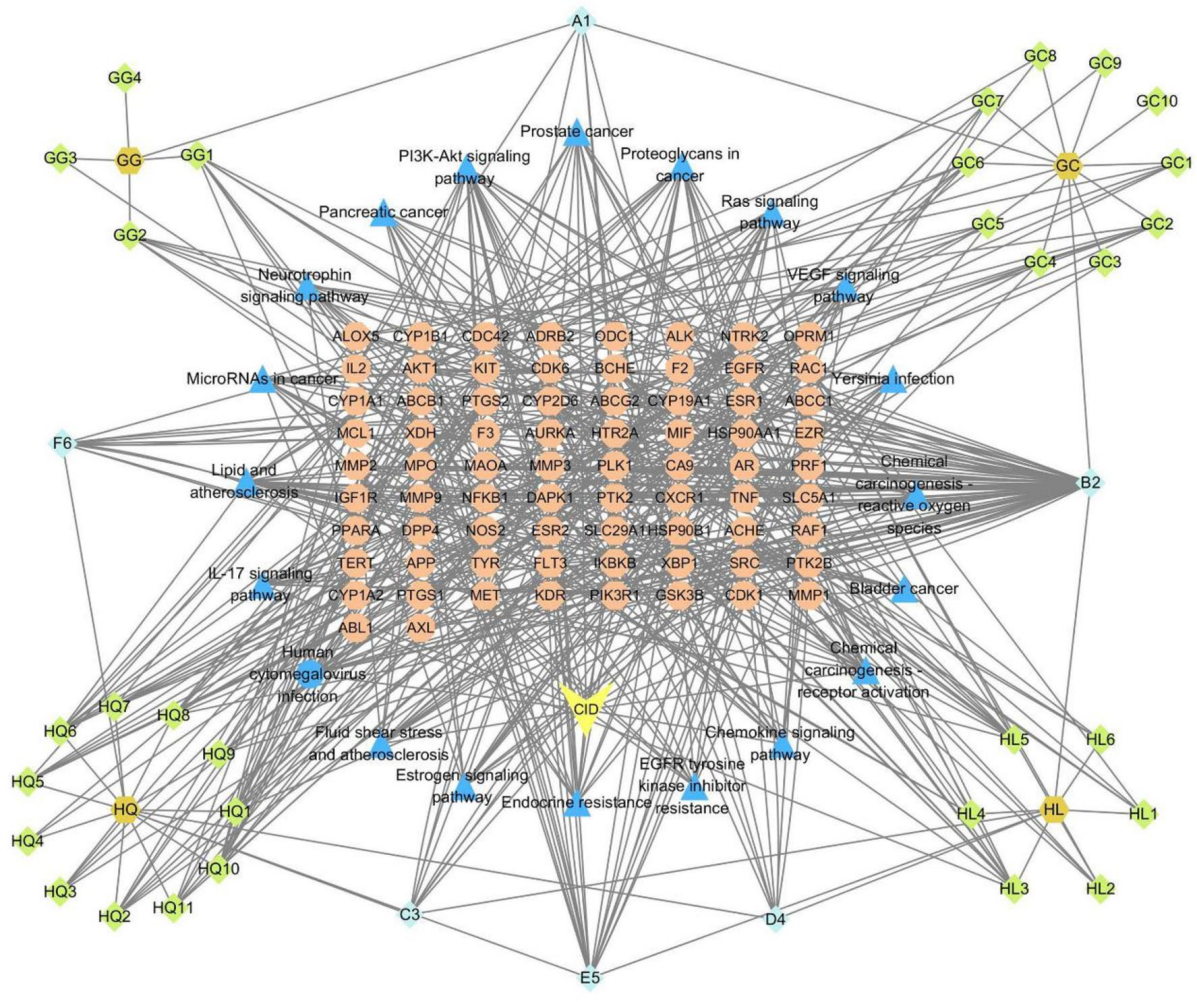
Network of compound-target-pathway–disease pathway. The pale orange hexagon nodes represent the four botanicals in GQD standard decoction. The green rhombus nodes represent the active compound contained in GQD standard decoction. The pale green rhombus nodes represent the common active compound contained. The orange hexagon nodes represent the protein. The blue triangle nodes represent the signal path. The yellow v-shaped node represents disease. Edges represent the interaction between them

Based on the proteins and pathways enrichment analysis, further constructing the C-T-D network of the GQD standard decoction’s active compounds. The C-T-D network was constructed by using Cytoscape software (Fig. 4), which was composed of 135 nodes (4 traditional Chinese medicines, 37 compounds, 20 signal pathways, 74 proteins) and 576 edges. The average degree value of core proteins was 8.3. The Quercetin (B2, degree = 62) from GC and HL, Acacetin (E5, degree = 20) from HQ and HL, Puerarin (HL4, degree = 12) from HL, Berberrubine (HL5, degree = 12) from HL, Worenine (HL3, degree = 11) from HL, Wogonin (HQ10, degree = 11) from HQ were discovered to be relatively high-degree active compounds, which suggested that the six compounds in CPT-11-induced diarrhea play more important roles.

### Effects of GQD standard decoction on NCM460 Cells Induced by SN-38

**Fig. 5:**
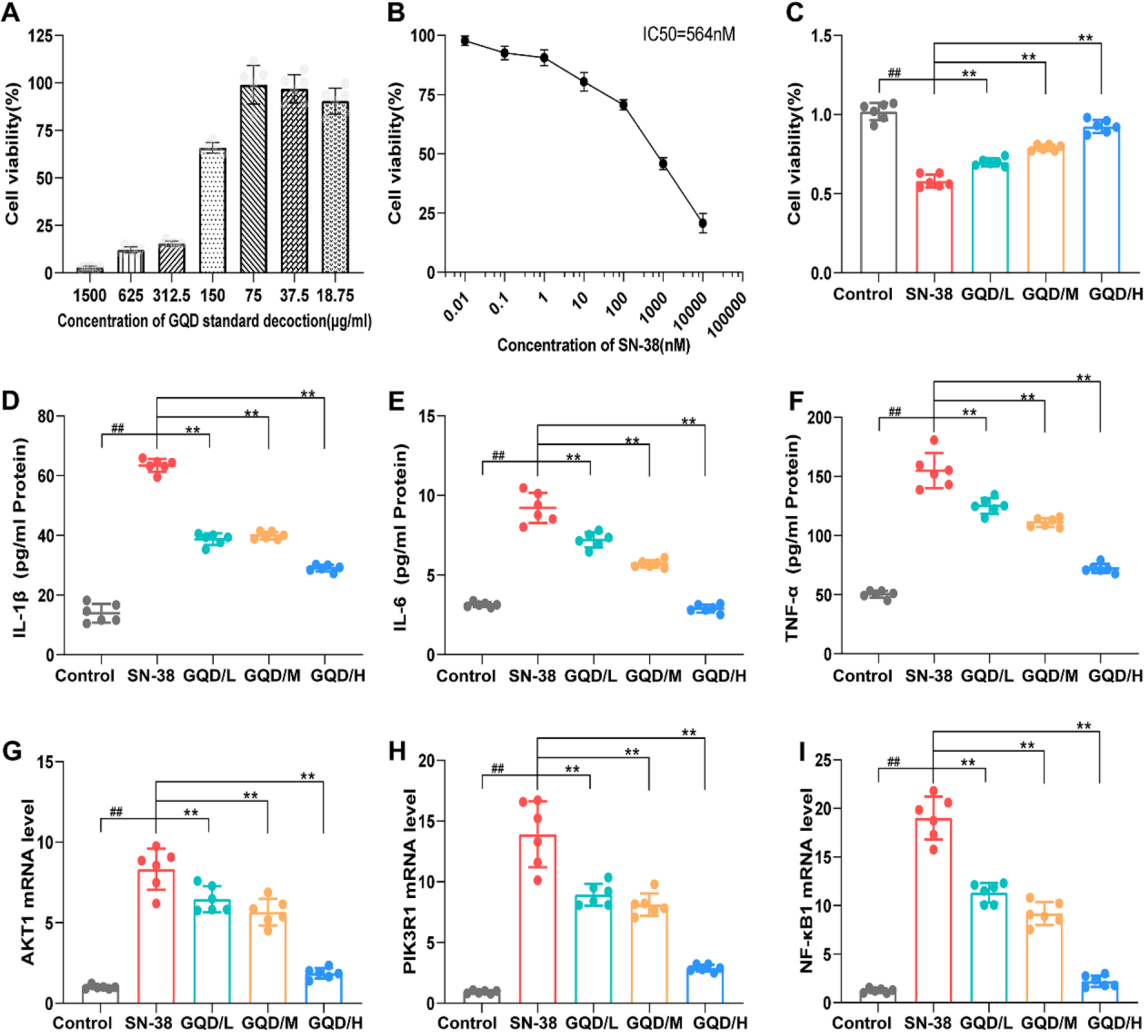
In vitro therapeutic outcomes of GQD standard decoction against CPT-11-induced CID. **A** Cell viability of GQD standard decoction in NCM 460 cells. **B** Antiproliferative activities of SN-38 in NCM 460 cells. **C** Cell viability of different doses of GQD standard decoction in NCM460 cells. Effects of GQD standard decoction on the amounts of proinflammatory [IL-1β (**D**), IL-6 (**E**), and TNF-α (**F**)] factors produced by NCM 460 cells. mRNA levels of AKT1 (**G**), PIK3R1 (**H**), and NF-κB1 (**I**). Data represented as mean ± SD. ##P < 0.01 vs. the control group; **P < 0.01 vs. the various treatment groups

In our previous study, we found that the ethanol extract and pill formulation of GQD can alleviate CPT-11-induced diarrhea in mice. It is known that SN-38 is active derivative of CPT-11, which is responsible for CPT-11 efficacy and toxicity [24]. And increased intestine SN-38 exposure is link to severe cytotoxicity. Hence, we employed SN-38 to cause damage and inflammation in colonic epithelial cells in vitro. First, seven concentrations of GQD standard decoction on the proliferation of NCM460 cells was assessed, and the nontoxic dose was screened. As shown in Fig. 5A, each preparation of GQD standard decoction showed concentration dependence. And GQD standard decoction showed various degrees of cytotoxicity in NCM460 cells when its concentrations above 75 µg/mL, therefore, the concentrations of 18.75, 37.5 and 75 µg/mL of GQD standard decoction was choosed for all subsequent cellular experiments. Meanwhile, the cytotoxicity of SN-38 in NCM460 cells was determined. As shown in Fig. 5B, the IC50 values of SN-38 in NCM460 cells were 564 nM, and 500 nM of SN-38 was employed to activate the cell damage model, which reduced the viability of cells about 40%. And Fig. 5C demonstrates that all GQD standard decoction ameliorated the cell damage induced by SN-38, in which GQD/H (75 µg/mL) exhibited the maximum cell viability in NCM460 cells stimulated by SN-38. Furthermore, Fig. 5D–F demonstrates that GQD standard decoction decreased IL-6, IL-1β, and TNF-α induced by SN-38. GQD/H exhibited much higher capacity than GQD/L and GQD/M, which was in accordance with its prominent effect on alleviating cell viability suppression. Furthermore, the PIK3R1, AKT1, NF-κB1was identified as core proteins using PPI network analysis. It is known, NF-κB signaling pathway is one of the important downstream pathways for PI3K/AKT signaling pathway, so we evaluated relative AKT1, PIK3R1, and NF-κB1 mRNA expression in NCM460 cells (Fig. 5G–I). The mRNA expression level of AKT1, PIK3R1, and NF-κB1 decreased in the GQD standard decoction groups. This finding indicated that GQD standard decoction effectively alleviated the colonic epithelial injury, related inflammatory levels and inhibited activation of PI3K/AKT/NF-κB pathway in NCM460 cells in vitro.

### GQD standard decoction exerts therapeutic effects against CPT-11-induced diarrhea in vivo

**Fig. 6:**
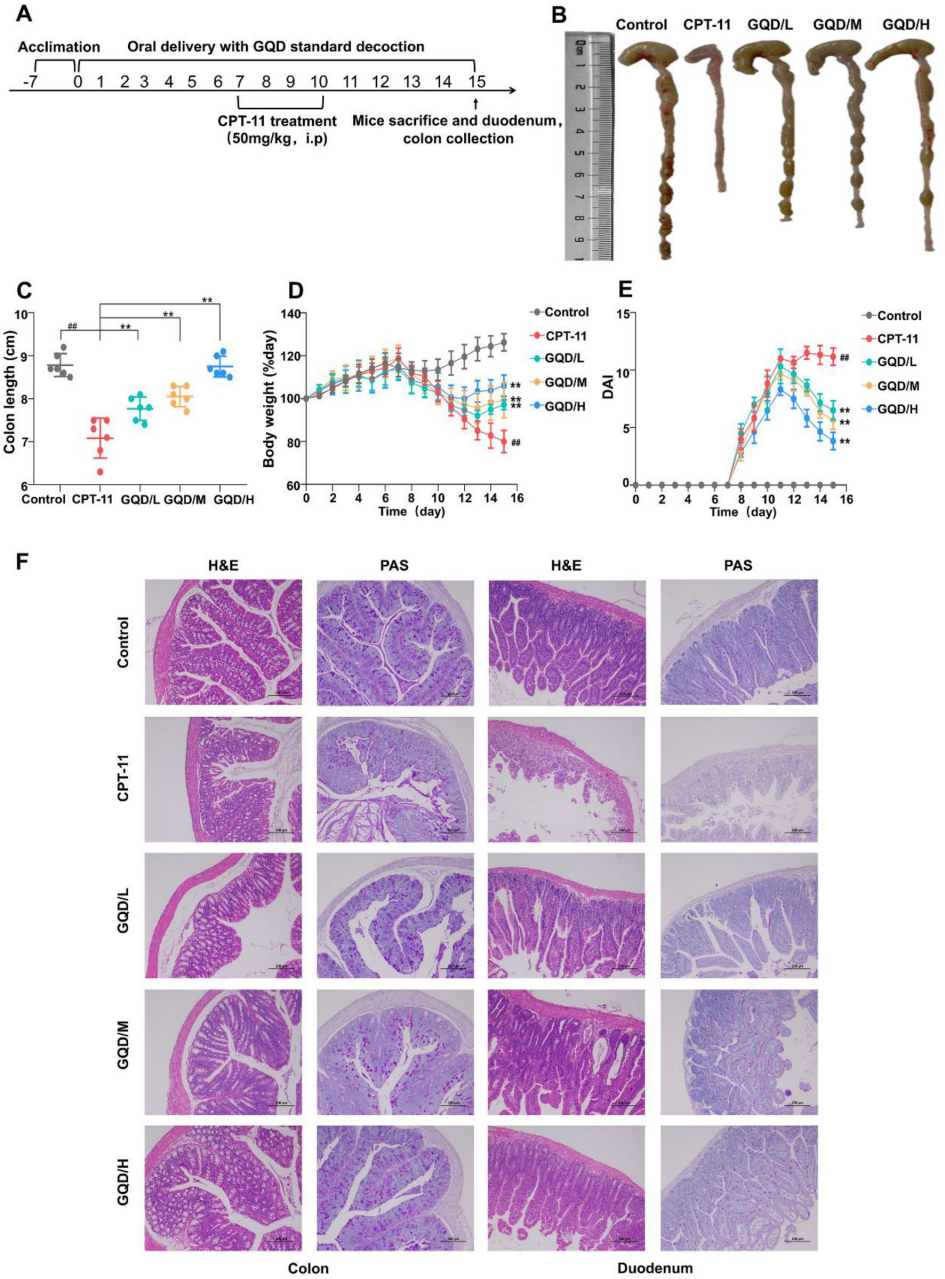
GQD standard decoction against CPT-11-induced CID in mice. **A** Modeling and treatment cycle. **B** Photographs of colons in various treatment groups. **C** Colon length of various treatment groups. **D** Body weight of various treatment groups. **E** DAI of various treatment groups. **F** Colon and duodenal sections were stained with H&E and PAS. The values represent the mean ± SD. ##P < 0.01 vs. the control group; **P < 0.01 vs. the GQD standard decoction group

To verify the therapeutic effects of GQD standard decoction on CPT-11-induced diarrhea treatment, we induced a diarrhea model by intraperitioneal injection of CPT-11 [4, 5], and the GQD standard decoction groups' therapeutic effect were also compared with the CPT-11 group. The animal modeling and treating was shown in Fig. 6A. The mice treated with GQD standard decoction groups had notably reduced colonic shortening, and the GQD-H had a more effective therapeutic impact than GQD-L and GQD-M (Fig. 6B and C). For the body weight (Fig. 6D), the weight loss of CPT-11 group have been effectively improved after GQD standard decoction treatment, and the GQD-H group have the most noticeable protective impact. Similar differences were observed for DAI scores [25]. As shown in Fig. 6E, DAI of the GQD standard decoction groups decreased was lower than that of the CPT-11 group. Meanwhile, DAI of the GQD-H group was much lower than the other groups of GQD standard decoction [26]. H&E staining of the intestines of mice in CPT-11 group revealed severe damage both the duodenum and colon (Fig. 6F), villous shortening, crypt disintegration, apoptosis, vacuolization of cells, oedema, and infiltration of polymorphonuclear cells were found in the duodenum and colon compared to the control tissue. Interestingly, all these defects were less noticeable in GQD standard decoction-treated mice, and the GQD-H group have the most noticeable protective impact on the duodenum and colon epithelial cells. Normal gut function depends on a balance in mucus production [26]. Accordingly, we measured the levels of mucin-liken glycoproteins in the duodenum and colon by PAS staining, as shown in Fig. 6F. The number of mucin-liken glycoproteins in the CPT-11 group was significantly lower than in the control group. However, such changes were been inhibit in the duodenum and colon tissues obtained from the GQD standard decoction groups, and the GQD-H group had the best protective effect, which was consistent with H&E staining results.

### GQD standard decoction prevents tight junction alterations in CPT-11-exposed mice

**Fig. 7:**
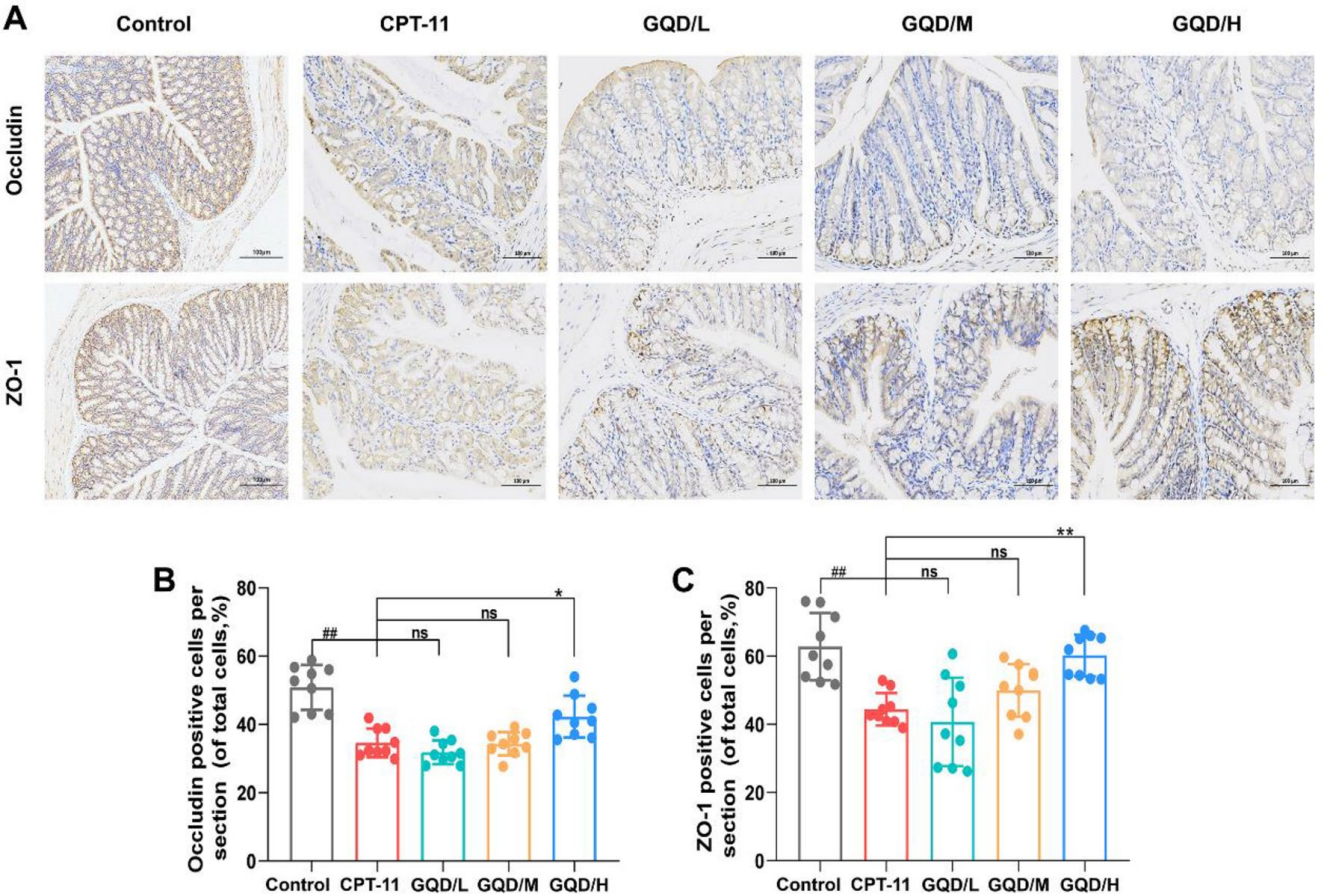
GQD standard decoction attenuated intestinal barrier function of CPT-11-induced CID in mice. **A** Immunohistochemistry staining of Occludin and ZO-1 protein. Representative images of Occludin (**B**) and ZO-1 (**C**) protein level in colon determined by immunohistochemistry. The values represent the mean ± SD. ##P < 0.01 vs. the control group; **P < 0.01, *P < 0.05 vs. the GQD standard decoction group

The intestinal barrier can inhibit dangerous substances from accessing other tissues or blood circulation through gut mucosa [27]. Actin–globulin ring surrounding TJ proteins contracts, transmembrane proteins such as Occludin and ZO-1 are degraded, which leads to intestinal mucosa injury [28, 29]. Immunohistochemistry analysis of colon samples for the tight junction proteins are shown in Fig. 7. In the CPT-11 group, the expression of Occludin and ZO-1 reduced considerably, indicating intestinal barrier impairment. And the GQD-H significantly enhanced Occludin and ZO-1 expression than other treatments, indicating it can protect the intestinal barrier.

### Experimental confirmation of putative proteins and GQD standard decoction suppresses colonic inflammatory levels

**Fig. 8:**
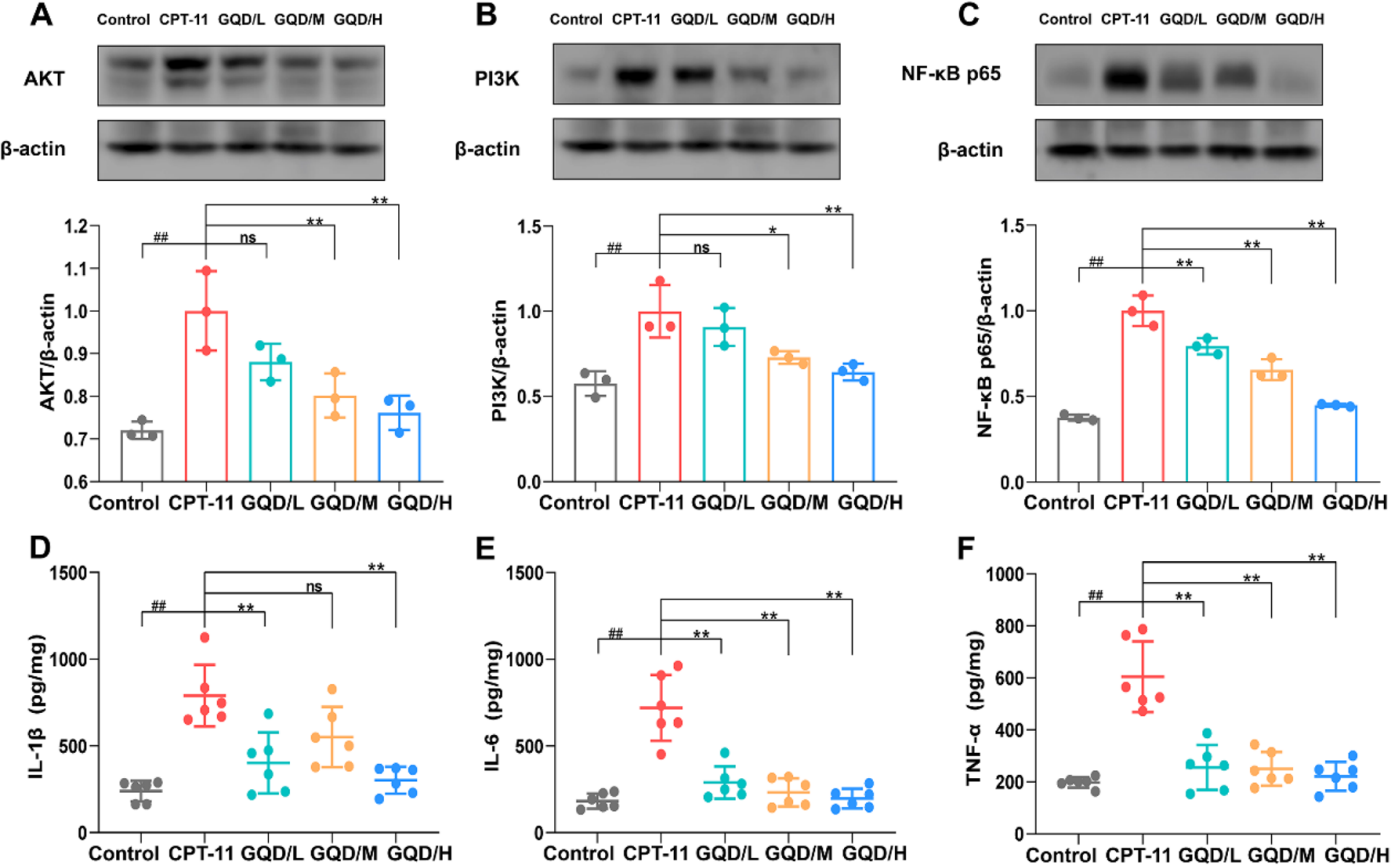
Potential proteins and GQD standard decoction for the treatment of inflamed colon in mice. Expression of AKT (**A**), PI3K (**B**), and NF-κB p65 (**C**) Western blots detected protein expression changes in colon tissues normalized to -actin. The levels of IL-1β (**D**), IL-6 (**E**), and TNF-α (**F**) in colon tissues were measured by ELISA. Data represented as mean ± SD. ##P < 0.01 vs. the control group; **P < 0.01 vs. the various treatment groups

According to network pharmacological study, the against CPT-11-induced diarrhea effect of GQD standard decoction might be strongly connected with PI3K/AKT/NF-κB pathway, we further confirmed the hypotheses regarding these putative proteins and signaling pathways. Including three proteins—PI3K, AKT1, and NF-κB p65. As shown in Fig. 8A–C, PI3K, AKT1, and NF-κB p65 protein levels in CPT-11 group were significantly higher (P < 0.01) than control group. However, the administration of GQD standard decoction reversed the increase in PI3K, AKT1, and NF-κB p65 levels.

It is known, Activated NF-κB pathway stimulates the expression of pro-inflammatory factors like TNF-α, IL-1β, IL-6 [30, 31], TNF and IL-6 cause early damage to connective tissue and endothelium, results in the loss of epithelial basal cells [32]. To find out how GQD standard decoction affects inflammation in colonic tissue, we measured concentrations of TNF-α, IL-1β, and IL-6 in colon tissue. As shown in Fig. 8D–F, comparing with the control group, TNF-α, IL-1β, and IL-6 were significantly increased by CPT-11 (P < 0.01). After GQD standard decoction treatment, the expression of these cytokines was obviously lower than in the CPT-11 group (P < 0.01), and GQD-H group had a better efffect than other groups.

## Discussion

CPT-11-induced diarrhea is an enormous toxicity to its cancer treatment, For about 40% of patients, such toxicity can result in treatment withdrawal or lower dose intensity for 40% of patients, which limits the drug 's efficacy [32–34] and may even lead to death. We have proved that the ethanol extract of GQD and extract fraction and the pill preparation of Gegen Qinlian can alleviate CPT-11-induced diarrhea in mice. However, the effect of GQD standard decoction with ancient records on CPT-11-induced diarrhea are not well understood. In this study, LC–MS analysis integrated with network pharmacology identified the underlying mechanisms of 37 effective chemical compounds of GQD standard decoction on CID. In addition, we verified the therapeutic role and mechanism of GQD standard decoction on CPT-11-induced diarrhea in vivo and vitro. We found that GQD standard decoction treatment significantly improved CPT-11-induced intestinal mucositis, including intestinal mucosal barrier, inflammatory factors, and signaling pathway expression. Moreover, GQD standard decoction suppressed SN-38 induced the inflammatory factors production and PI3K/AKT/NF-κB activation in NCM460 cells. These findings help with CPT-11-induced diarrhea treatment.

Although the exact mechanism of CPT-11-induced diarrhea pathogenesis is unknown, various evidence shows that alterations in the inflammation process, histopathological and enteric barrier function alterations, are the important factors of CPT-11-induced diarrhea pathogenesis [35]. In this study, we first used LC–MS dissected chemical constituents in GQD standard decoction, a total of 130 compounds were identified from GQD standard decoction, the probable anti-CID mechanism of the 37 active components in GQD standard decoction was anticipated using a network pharmacology study. The chemical components within GQD standard decoction can be complex and varied, 37 active compounds identified from GQD standard decoction mainly including flavonoids, alkaloids, carboxylic acids, coumarins, saponins. Guan et al. demonstrated that Glycyrrhizin and glycyrrhetinic acid can selectively inhibited cyclooxygenase-2 (COX-2) activity to alleviate diarrhea [36, 37]. And the flavonoid derived from scutellaria could be dose-dependently attenuated CID via suppression of up-regulation of inflammatory cytokines such as TNF-α, IL-1β, and TGF-β [38]. And the alkaloids such as berberine, palmatine, coptisine of *Coptis chinens*is has been widely demonstrated for treating gastrointestinal diseases, its anti-inflammatory effects involved in MAPK, NF-κB, and PPAR-γ signaling pathway regulation are highlighted in the treatment of gastrointestinal disorders [7]. Among the 37 active ingredients, there are still some main chemical components such as quercetin, puerarin, daidzin, daidzein and acacetin have also been confirmed to have good anti-inflammatory. Therefore, further investigations should focus on characterization of bioactive compounds responsible for the protective effects against CID, the quantification and mechanisms of action should be in-depth studied. The network pharmacology analysis demonstrated that GQD standard decoction mainly interferes with the occurence and development of CID through the inflammatory and apoptosis signaling pathways, such as PI3K/AKT, Ras, EGFR and NF-κB signaling pathway, which is intimately related to CID pathogenesis [39, 40]. And PIK3R1, AKT1, NF-κB1was identified as core proteins using PPI network analysis. It is well known that one of the significant downstream pathways for the PI3K/AKT signaling pathway is the NF-κB signaling pathway. Excitation of the PI3K/AKT pathway stimulates IKK, which activates NF-κB RelA/p65 [41, 42]. Therefore, we wonder that GQD standard decoction may directly regulate PI3K/AKT/NF-κB to improve the inflammatory response and promote the recovery of damaged intestinal tissue after CPT-11 treatment. We used western blotting to measure the expression of PI3K and AKT and NF-κB in colon tissues, GQD standard decoction reduced the expression of PI3K and AKT and NF-κB in control group. In NCM460 cells in vitro, GQD standard decoction reduced the upregulation of PIK3R1, AKT1, NF-κB1 induced by SN-38. These are consistent with network pharmacology analysis, further demonstrating that PI3K/AKT/NF-κB signaling pathway plays an important role in GQD standard decoction against CPT-11-induced diarrhea.

As important pathogenesis of CPT-11-induced diarrhea, intestinal mucosal barrier alterations is also the hallmark [43]. The colonic SN-38 produces severe damage to the enteric system, since it is capable of killing enterocytes and can result in delayed diarrhea [44]. Tight junctions are multiple protein complexes which regulate intestinal permeability controlling the movement of fluids, nutrients, microbes and toxins across the epithelia. It is known that ZO-1 and Occludin play critica roles in maintaining tight junctions integrity and the mucosal barrier function [45], consistent with prior results [46], CPT-11 therapy reduced the expression of ZO-1 and Occludin [47]. Conversely, treatment with GQD standard decoction maintained the levels and position of both proteins in a range similar to mice in the control group, proving the potential of GQD standard decoction to protect intestinal mucosa from the effects of mucositis. Our finding also shown that SN-38 could harm mucus layers, the mucus layer is a glycosylated hydrated gel generated by mucin proteins that separates intestinal epithelium from intestinal contents [48], as shown by PAS staining, indicating that CPT-11 depleted goblet cells in the colon’s mucin-like glycoproteins, which was also verified by recent research [26], however, treatment with GQD standard decoction greatly conserved the quantity of mucin in the colon. This set of data offers additional evidence for the protective effects of GQD standard decoction on the intestine.

Among inflammation, CPT-11 and SN-38 attacked the initial epithelial NF-κB signaling pathway leading to production of proinflammation factors. TNF-α and IL-6 cause early damage in connective tissue and activate matrix metalloproteinases 1 and 3 in intestinal epithelial cells and lamina propria [49], leading in damage and death of epithelial basal cell [50], which is a severe and critical symptoms of CPT-11-induced diarrhea. And our finding were also supported by in vitro inflammatory cell model and CPT-11-induced animal models, according to the result, as compared with the control group, CPT-11 significantly increased levels of TNF-α, IL-1β, and IL-6, however, GQD standard decoction ameliorated this inflammatory process, as seen in NCM 460 cells and colon.

## Conclusions

In this study, using an integrated strategy, the molecular mechanism of 37 active ingredientsm in GQD standard decoction against CPT-11-induced diarrhea were demonstrated. UPLC-MS/MS analysis revealed 37 effective chemical compounds of GQD standard decoction and used for subsequent network pharmacology analysis. And the core proteins and pathway were validated by experiment. We demonstrated that GQD standard decoction could suppress inflammatory and intestinal mucosal barrier alterations via PI3K/AKT/ NF-κB signaling pathway, and it were further verified in vivo and vitro experiments. This data establishes the groundwork for particular molecular mechanism of GQD standard decoction active components. Furthermore, this research can serve as a new foundation for the TCM therapy of CID. However, the clinical effect of GQD standard decoction on CPT-11-induced diarrhea, has to be studied further.

## Supplementary Information

**Additional file 1**: **Material S1.** The HPLC determination conditions. **Table S1.** A total of 130 compounds in GQD standard decoction. **Table S2.** Contents of 11 chemicals in fifteen batches of GQD. **Table S3.** The proteins of active compounds in GQD standard decoction. **Table S4.** The 74 potential proteins of GQD standard decoction for the treatment of CID. **Table S5.** KEGG enrichment analysis results.

**Additional file 2**: **Figure S1.** Mass spectrum chromatograms of reference standards.

**Additional file 3**: **Material S2.** Disease Protein genes of CID found from the Genecards database and the OMIM database.

## Data Availability

The data used to support the findings of this study are included within the article and the supplementary information files.

## Abbreviations:

ADME: Absorption, distribution, metabolism and excretion
AKT1: AKT serine/threonine kinase 1
BP: Biological processes
CC: Cellular components
DL: Drug-likeness
ELISA: Enzyme-linked immunosorbent assay
GAPDH: Glyceraldehyde 3-phosphate dehydrogenase
GO: Gene ontology
KEGG: Kyoto encylopedia of genes and genomes
MF: Molecular function
NF-κB p65: Nuclear factor-kappa B P65
OB: Oral bioavailability
OMIM: Online mendelian inheritance in man
PI3K: Phosphatidylin-ositol-3-kinase
PPI: Protein–protein interaction
STRING: Search tool for the retrieval of interacting genes/proteins
TCM: Traditional Chinese medicine
TCMSP: Traditional Chinese medicine systems pharmacology database
