# Supplementary material for: Gegen Qinlian standard decoction alleviated irinotecan-induced diarrhea via PI3K/AKT/NF-κB axis by network pharmacology prediction and experimental validation combination
Source: Chin Med. 2023 Apr 27;18:46. doi: 10.1186/s13020-023-00747-3 (PMC10134581; doi:10.1186/s13020-023-00747-3)
Supplement: Supplementary file 1 — Additional file 1: Material S1. The HPLC determination conditions. Table S1. A total of 130 compounds in GQD standard decoction. Table S2. Contents of 11 chemicals in fifteen batches of GQD. Table S3. The proteins of active compounds in GQD standard decoction. Table S4. The 74 potential proteins of GQD standard decoction for the treatment of CID. Table S5. KEGG enrichment analysis results. [file 13020_2023_747_MOESM1_ESM.doc]

**Gegen Qinlian standard decoction alleviated** **irinotecan-induced diarrhea via PI3K/AKT/NF-κB axis by network pharmacology prediction and experimental validation combination**

Jiamei Chen^1^, Min Li^1^, Rong Chen^1^, Ziyi Xu^1^, Xiaoqin Yang^1^, Huan Gu^1^, Lele Zhang^2^, Chaomei Fu^1,*^, Jinming Zhang^1,*^, Yihan Wu ^1,*^

*^1^State Key Laboratory of southwestern Chinese Medicine Resources, Pharmacy School, Chengdu University of Traditional Chinese Medicine, Chengdu 611137, China*

*^2^School of Medicine, Chengdu University, Chengdu 610106, China*

**Material S1.** The HPLC determination conditions.

**Table S1.** A total of 130 compounds in GQD standard decoction.

**Table S2.** Contents of 11 chemicals in fifteen batches of GQD.

**Table S3.** The proteins of active compounds in GQD standard decoction.

**Table S4.** The 74 potential proteins of GQD standard decoction for the treatment of CID.

**Table S5.** KEGG enrichment analysis results.

**Material S1.** The HPLC determination conditions.

HPLC instrumentation and conditions: Quantitative analysis were performed using UltiMate 3000 HPLC instrument (ThermoFisher, USA) with a DAD 3000 detector, a ternary pump of SR3000 Solvent Rack, a WPS-3000SL autosampler, a TCC-3000SD column temperature controller, and a workstation of Chromeleon 7.2.

The puerarin, daidzin, daidzein separation was performed on a Thermo Hypersil Gold C18 column (250 mm×4.6 mm, 5 μm). The mobile phase consisted of purified water (solvent A) and methanol (solvent B) at a flow rate of 1 mL/min, which followed a gradient program of 0–20 min, 25% A; 20–30 min, 25–45% B; 30–40 min, 45–60% B; 40–50 min, 60–75% B. The column temperature was maintained at 30 °C while the autosampler temperature was maintained at 4 °C with 10 μL of sample solution being injected. The detection wavelength of DAD was monitored at 250 nm.

The baicalin, wogonin, baicalein, wogonoside was performed on a Thermo Hypersil Gold C18 column (250 mm×4.6 mm, 5 μm). The mobile phase consisted of 0.1% formic acid (solvent A) and acetonitrile (solvent B) at a flow rate of 1 mL/min, which followed a gradient program of 0–10 min, 25-30% B; 10–30 min, 30–50% B; 30–35 min, 50–100% B; 35–40 min, 100–25% B. The column temperature was maintained at 30 °C while the autosampler temperature was maintained at 4 °C with 10 μL of sample solution being injected. The detection wavelength of DAD was monitored at 275 nm.

The coptisine, berberine, palmatine was performed on a Thermo Hypersil Gold C18 column (250 mm×4.6 mm, 5 μm). The mobile phase consisted of 0.05 mol·L-1 potassium dihydrogen phosphate (Adjust pH to 3.0 with phosphoric acid) (solvent A) and acetonitrile (solvent B) at a flow rate of 1 mL/min. The column temperature was maintained at 30 °C while the autosampler temperature was maintained at 4 °C with 10 μL of sample solution being injected. The detection wavelength of DAD was monitored at 346 nm.

The liquiritin was performed on a Thermo Hypersil Gold C18 column (250 mm×4.6 mm, 5 μm). The mobile phase consisted of 0.1% phosphoric acid (solvent A) and acetonitrile (solvent B) at a flow rate of 1 mL/min, which followed a gradient program of 0–3 min, 19% B; 3–20 min, 19–26% B; 20–22 min, 26–40% B; 22–24 min, 40% B; 34–36 min, 40-19% B; The column temperature was maintained at 30 °C while the autosampler temperature was maintained at 4 °C with 10 μL of sample solution being injected. The detection wavelength of DAD was monitored at 237 nm.

**Table S1. A total of 130 compounds in GQD standard decoction.**

| No. | t_R_  (min) | Compound | Molecular formula | Extracted ions | Measured value | Type | Source |
| --- | --- | --- | --- | --- | --- | --- | --- |
| 1 | 2.31 | Licochalcone A | C_21_H_22_O_4_ | [M+H]^+^ | 339.1591 | flavonoids | GC |
| 2 | 2.42 | Sucrose | C_12_H_22_O_11_ | [M+H]^+^ | 343.1234 | glycosides | GG |
| 3 | 2.87 | Malonicacid | C_3_H_4_O_4_ | [M-H]^-^ | 103.0036 | carboxylic acids | GG |
| 4 | 2.92 | Quinicacid | C_7_H_12_O_6_ | [M-H]^-^ | 191.0561 | carboxylic acids | HL |
| 5 | 2.92 | D-saccharicacid | C_6_H_10_O_8_ | [M-H]^-^ | 209.0302 | carboxylic acids | HQ |
| 6 | 3.58 | Malicacid | C_4_H_6_O_5_ | [M-H]^-^ | 133.0136 | carboxylic acids | HQ |
| 7 | 3.62 | P-coumaricacid | C_9_H_8_O_3_ | [M+H]^+^ | 165.0546 | carboxylic acids | GG |
| 8 | 5.09 | Citricacid | C_6_H_8_O_7_ | [M-H]^-^ | 191.0197 | carboxylic acids | HQ |
| 9 | 8.23 | Hydroxybenzoateglycoside | C_13_H_16_O_8_ | [M-H]^-^ | 299.0778 | others | GC |
| 10 | 10.56 | Glucosylisomaltol | C_12_H_16_O_8_ | [M+H]^+^ | 289.0918 | others | GG |
| 11 | 10.95 | L-Tryptophan | C_11_H_12_N_2_O_2_ | [M+H]+/  [M-H]^-^ | 205.0971/  203.0826 | others | HL,  GG |
| 12 | 12.01 | Licoisoflavone B | C_20_H_16_O_6_ | [M-H]^-^ | 351.0877 | flavonoids | GC |
| 13 | 12.16 | Vanillicacid | C_8_H_8_O_4_ | [M-H]^-^ | 167.0349 | carboxylic acids | HQ,  HL |
| 14 | 12.93 | Magnoflorine | C_20_H_23_NO_4_ | [M+H]^+^ | 342.1699 | alkaloids | HL |
| 15 | 16.35 | Chlorogenicacid | C_16_H_18_O_9_ | [M-H]^-^ | 353.0878 | carboxylic acids | HL |
| 16 | 19.51 | Puerarin-4'-O-β-D-glucopyranoside | C_27_H_30_O_14_ | [M-H]^-^ | 577.1562 | flavonoids | GG |
| 17 | 22.69 | Berlambine | C_20_H_17_NO_5_ | [M+H]^+^ | 352.1179 | alkaloids | HL |
| 18 | 23.62 | Narcissoside | C_28_H_32_O_16_ | [M-H]^-^ | 623.1604 | glycosides | GC |
| 19 | 23.64 | Violanthin | C_27_H_30_O_14_ | [M+H]^+^ | 579.1718 | flavonoids | GC |
| 20 | 23.64 | Daidzein-4',7-Diglucoside | C_27_H_30_O_14_ | [M+H]^+^ | 579.1708 | flavonoids | GG |
| 21 | 25.04 | Apigenin-7-Glucoside | C_21_H_20_O_10_ | [M-H]^-^ | 431.0983 | flavonoids | HQ |
| 22 | 25.08 | 3'-Hydroxypuerarin | C_21_H_20_O_10_ | [M+H]^+^ | 433.1129 | flavonoids | GG |
| 23 | 25.12 | P-Hydroxybenzylmalonicacid | C_10_H_10_O_5_ | [M-H]^-^ | 209.0459 | others | GC |
| 24 | 25.16 | 5-Ethoxyisophthalicacid | C_10_H_10_O_5_ | [M-H]^-^ | 209.0461 | others | GC |
| 25 | 25.50 | Berberrubine | C_19_H_16_NO_4_ | [M+H]+/  [M-H]^-^ | 323.1152/321.1006 | alkaloids | HL |
| 26 | 25.52 | Neochlorogenicacid | C_16_H_18_O_9_ | [M-H]^-^ | 353.0878 | carboxylic acids | HL |
| 27 | 28.04 | Uralneoside | C_12_H_14_O_8_ | [M-H]^-^ | 285.0611 | saponins | GC |
| 28 | 30.81 | Isorhamnetin-7-O-Rhagluacid | C_28_H_30_O_17_ | [M-H]^-^ | 637.1417 | alkaloids | HQ |
| 29 | 30.88 | Groenlandicine | C_19_H_16_NO_4_ | [M+H]^+^ | 323.1152 | alkaloids | HL |
| 30 | 30.91 | Columbamine | C_20_H_20_NO_4_ | [M+H]^+^ | 339.1465 | alkaloids | HL |
| 31 | 31.33 | Puerarin | C_21_H_20_O_9_ | [M-H]^-^ | 415.1034 | flavonoids | GG |
| 32 | 31.88 | Coptisine | C_19_H_14_NO_4_ | [M+H]^+^ | 321.0995 | alkaloids | HL |
| 33 | 32.55 | 3'-Methoxypuerarin | C_22_H_22_O_10_ | [M-H]^-^ | 445.1140 | flavonoids | GG |
| 34 | 33.47 | Berberine | C_20_H_18_NO_4_ | [M+H]^+^ | 337.1308 | alkaloids | HL |
| 35 | 33.47 | Epiberberine | C_20_H_18_NO_4_ | [M+H]^+^ | 337.1308 | alkaloids | HL |
| 36 | 34.07 | Mirificin | C_26_H_28_O_13_ | [M+H]^+^ | 549.1602 | flavonoids | GG |
| 37 | 34.56 | Palmatine | C_21_H_22_NO_4_ | [M+H]^+^ | 353.1621 | alkaloids | HL |
| 38 | 35.25 | Daidzin | C_21_H_20_O_9_ | [M+H]^+^ | 417.1182 | flavonoids | GG |
| 39 | 35.25 | Genistein | C_15_H_10_O_5_ | [M-H]^-^ | 269.0455 | flavonoids | GC,  GG |
| 40 | 35.45 | Worenine | C_20_H_15_NO_4_ | [M+H]^+^ | 334.1073 | alkaloids | HL |
| 41 | 35.88 | Noroxyhydrastinine | C_10_H_9_NO_3_ | [M+H]^+^ | 192.0655 | alkaloids | HL |
| 42 | 35.89 | Enol-phenylpyruvate | C_9_H_8_O_3_ | [M-H]^-^ | 163.0400 | others | GG |
| 43 | 36.37 | Calycosin-7-glucoside | C_22_H_22_O_10_ | [M+H]^+^ | 447.1258 | flavonoids | GC,  GG |
| 44 | 36.37 | 3'-Methoxydaidzein | C_16_H_12_O_5_ | [M+H]^+^ | 285.0768 | flavonoids | GG |
| 45 | 36.37 | 3’-Methoxydaidzin | C_22_H_22_O_10_ | [M+H]^+^ | 477.1285 | flavonoids | GG |
| 46 | 37.77 | Tectoridin | C_22_H_22_O_11_ | [M+H]^+^ | 463.1234 | flavonoids | GG |
| 47 | 37.85 | 8-Methoxyformononetin | C_23_H_24_O_10_ | [M+H]^+^ | 461.1429 | others | GG |
| 48 | 37.85 | Moslosooflavone | C17H14O5 | [M+H]^+^ | 299.0914 | flavonoids | HQ |
| 49 | 37.86 | Puerosides | C_29_H_34_O_14_ | [M-H]^-^ | 605.1875 | flavonoids | GG |
| 50 | 38.25 | 3′-Hydrogenatedpuerarinxyloside | C_26_H_28_O_14_ | [M+H]^+^ | 565.1552 | others | GG |
| 51 | 38.26 | Genistein8-C-Glucoside | C_26_H_28_O_14_ | [M-H]^-^ | 563.1413 | glycosides | GG |
| 52 | 39.87 | Liquiritinapioside | C_26_H_30_O_13_ | [M-H]^-^ | 549.1634 | flavonoids | GC |
| 53 | 39.87 | Isoliquiritinapioside | C_26_H_30_O_13_ | [M-H]^-^ | 549.1613 | flavonoids | GC |
| 54 | 39.87 | Liguiritigenin-7-O-Apiosyl-4’-O-Glucose | C_26_H_30_O_13_ | [M-H]^-^ | 549.1613 | glycosides | GC |
| 55 | 40.70 | Umbelliferone | C_9_H_6_O_3_ | [M+H]^+^ | 163.0389 | coumarins | GC |
| 56 | 40.87 | Genistin | C_21_H_20_O_10_ | [M+H]^+^ | 433.1192 | flavonoids | GG |
| 57 | 40.95 | Daidzein-4',7-o-Diglucoside | C_29_H_36_O_15_ | [M-H]^-^ | 623.1627 | flavonoids | GG |
| 58 | 41.41 | 5,7,8-Trihydroxyflavone | C_15_H_12_O_5_ | [M-H]^-^ | 271.0615 | others | HQ |
| 59 | 41.41 | Dihydrobaicalein | C_15_H_12_O_5_ | [M-H]^-^ | 271.0611 | flavonoids | HQ |
| 60 | 43.17 | Isocarthamidin-7-O-Glucuronide | C_21_H_20_O_12_ | [M-H]^-^ | 463.0881 | others | HQ |
| 61 | 43.17 | 2',3,5,6',7-Tetrahydroxyflavone-2'-O-Glucoside | C_21_H_20_O_12_ | [M-H]^-^ | 463.0881 | others | HQ |
| 62 | 43.54 | Carthamidin-7-O-Glucuronide | C_21_H_20_O_12_ | [M-H]^-^ | 463.0881 | others | HQ |
| 63 | 43.57 | Ononin | C_22_H_22_O_9_ | [M+H]^+^ | 431.1136 | flavonoids | GC,  GG |
| 64 | 43.57 | 4-Methoxypuerarin | C_22_H_22_O_9_ | [M-H]^-^ | 429.1191 | flavonoids | GG |
| 65 | 44.39 | Glycyroside | C_27_H_30_O_13_ | [M-H]^-^ | 561.1613 | glycosides | GC |
| 66 | 44.76 | Scuteamoenin | C_16_H_14_O_6_ | [M-H]^-^ | 301.0717 | flavonoids | HQ |
| 67 | 44.76 | Trihydroxy-Methoxyflavanone | C_16_H_14_O_6_ | [M-H]^-^ | 301.0717 | flavonoids | HQ |
| 68 | 44.82 | 5-Hydroxyliquiritin | C_21_H_22_O_10_ | [M-H]^-^ | 433.1140 | flavonoids | GC |
| 69 | 46.75 | 6”-O-Malonyldaidzin | C_24_H_22_O_12_ | [M+H]^+^ | 503.1184 | flavonoids | GG |
| 70 | 46.80 | 5,7,2',5-Tetrahydroxy-8,6-Dimethoxyflavone | C_17_H_14_O_8_ | [M+H]^+^ | 347.0761 | flavonoids | HQ |
| 71 | 46.80 | Ganhuangenin | C_17_H_14_O_8_ | [M+H]^+^ | 347.0766 | flavonoids | HQ |
| 72 | 47.09 | 5,7,4'-Trihydroxy-8-Methoxyflavone | C16H12O6 | [M-H]^-^ | 299.0561 | flavonoids | HQ |
| 73 | 47.29 | Isoliquiritin | C_21_H_22_O_9_ | [M-H]^-^ | 417.1191 | flavonoids | GC |
| 74 | 48.10 | Salicylicacid | C_7_H_6_O_3_ | [M-H]^-^ | 137.0247 | carboxylic acids | GG |
| 75 | 48.10 | 4-Hydroxybenzoicacid | C_7_H_6_O_3_ | [M-H]^-^ | 137.0244 | others | HQ |
| 76 | 48.63 | 7-Methoxyliguiritin | C_22_H_22_O_9_ | [M+H]^+^ | 431.1336 | others | GC |
| 77 | 48.63 | 8-Methoxy-5-O-Glucoside Flavonoid | C_22_H_22_O_9_ | [M+H]^+^ | 431.1336 | flavonoids | GC |
| 78 | 48.64 | Formononetin | C_16_H_12_O_4_ | [M-H]^-^ | 267.0662 | flavonoids | GC，GG |
| 79 | 48.64 | 7-Aldehydeglycyrrhizin | C_23_H_24_O_11_ | [M-H]^-^ | 475.1245 | others | GC |
| 80 | 48.99 | 6"-O-Acetyllicorice | C_23_H_24_O_10_ | [M-H]^-^ | 459.1322 | others | GC |
| 81 | 49.05 | 4-Methylumbelliferone | C_10_H_8_O_3_ | [M+H]^+^ | 177.0546 | coumarins | GC |
| 82 | 49.05 | 7-Methoxycoumarin | C_10_H_8_O_3_ | [M+H]^+^ | 177.0546 | coumarins | GC |
| 83 | 49.07 | Ferulicacid | C_10_H_10_O_4_ | [M-H]^-^ | 193.0506 | phenylpropanoids | GG |
| 84 | 49.35 | Moupinamide | C_18_H_19_NO_4_ | [M+H]^+^ | 314.1753 | alkaloids | HL |
| 85 | 49.81 | Licoflavonol | C_20_H_18_O_6_ | [M-H]^-^ | 353.1045 | flavonoids | GC |
| 86 | 50.35 | Quercetin | C_15_H_10_O_7_ | [M+H]^+^ | 303.0499 | flavonoids | GC,  HL |
| 87 | 50.59 | Isoliquiritigenin | C_15_H_12_O_4_ | [M-H]^-^ | 255.0663 | flavonoids | GC |
| 88 | 50.59 | Liquiritigenin | C_15_H_12_O_4_ | [M-H]^-^ | 255.0663 | flavonoids | GC |
| 89 | 50.96 | Liquiritin | C_21_H_22_O_9_ | [M+H]^+^ | 419.1336 | flavonoids | GC |
| 90 | 51.59 | 3,4-Dimethoxycinnamicacid | C_11_H_12_O_4_ | [M-H]^-^ | 207.0662 | others | HL |
| 91 | 51.83 | Licochalcone B | C_16_H_14_O_5_ | [M-H]^-^ | 285.0765 | flavonoids | GC |
| 92 | 53.06 | 3,2’-Dihydroxyflavone | C_15_H_10_O_4_ | [M+H]^+^ | 255.0651 | flavonoids | GG |
| 93 | 53.06 | Daidzein | C_15_H_10_O_4_ | [M+H]^+^ | 255.0651 | flavonoids | GG |
| 94 | 53.06 | Chrysin | C_15_H_10_O_4_ | [M+H]+/[M-H]^-^ | 255.0654/253.0506 | flavonoids | HQ |
| 95 | 53.80 | Biochanina | C_16_H_12_O_5_ | [M+H]^+^ | 285.0757 | flavonoids | GG |
| 96 | 53.82 | Wogonoside | C_22_H_20_O_11_ | [M+H]^+^ | 461.1084 | flavonoids | HQ |
| 97 | 53.82 | Wogonosideisomers | C_22_H_20_O_11_ | [M+H]^+^ | 461.1084 | flavonoids | HQ |
| 98 | 53.85 | Wogonin | C_16_H_12_O_5_ | [M+H]^+^ | 285.0763 | flavonoids | HQ |
| 99 | 53.85 | Oroxylina | C_16_H_12_O_5_ | [M+H]^+^ | 285.0760 | flavonoids | HQ |
| 100 | 53.88 | 3',4'-Dihydroxy-7-Methoxyisoflavone | C_16_H_12_O_5_ | [M-H]^-^ | 283.0612 | flavonoids | GC |
| 101 | 53.88 | Calycosin | C_16_H_12_O_5_ | [M-H]^-^ | 283.0613 | flavonoids | GC |
| 102 | 53.88 | Acacetin | C_16_H_12_O_5_ | [M+H]^+^ | 285.0757 | flavonoids | HQ |
| 103 | 54.25 | Panicolin | C_17_H_14_O_6_ | [M+H]^+^ | 315.0863 | flavonoids | HQ |
| 104 | 54.25 | Dihydroxy-Dimethoxyflavone | C_17_H_14_O_6_ | [M+H]^+^ | 315.0866 | flavonoids | HQ |
| 105 | 54.69 | 5-Hydroxy-2',6',7,8-Tetramethoxyflavone | C_18_H_16_O_8_ | [M-H]^-^ | 359.0772 | flavonoids | HQ |
| 106 | 55.04 | Martynoside | C_31_H_40_O_15_ | [M-H]^-^ | 651.2294 | phenylethanol glycosides | HQ |
| 107 | 55.04 | Isomartynoside | C_31_H_40_O_15_ | [M-H]^-^ | 651.2294 | phenylethanol glycosides | HQ |
| 108 | 57.12 | Dihydrobaicalin | C_21_H_20_O_11_ | [M+H]^+^ | 449.1078 | flavonoids | HQ |
| 109 | 57.14 | Naringenin | C_15_H_12_O_5_ | [M+H]^+^ | 273.0757 | flavonoids | GC |
| 110 | 57.14 | Carthamidin | C_15_H_12_O_6_ | [M+H]^+^ | 289.0707 | flavonoids | HQ |
| 111 | 57.14 | （2S)-5,7,2',6'-Tetrahydroxydihydroflavone | C_15_H_12_O_6_ | [M+H]^+^ | 289.0706 | flavonoids | HQ |
| 112 | 57.16 | Cynaroside | C_21_H_20_O_11_ | [M-H]^-^ | 447.0936 | flavonoids | HQ |
| 113 | 57.55 | Trihydroxy-Methoxyflavone-O-Glucuronide | C_22_H_20_O_12_ | [M-H]^-^ | 475.0881 | flavonoids | HQ |
| 114 | 57.92 | Tectoridin | C_22_H_22_O_11_ | [M+H]^+^ | 463.1198 | flavonoids | GG |
| 115 | 58.31 | Glycyrol | C_21_H_18_O_6_ | [M+H]^+^ | 367.1176 | coumarins | GC |
| 116 | 58.60 | Lupiwighteone | C_20_H_18_O_5_ | [M+H]^+^ | 339.1227 | flavonoids | GC |
| 117 | 58.65 | Scutellarin | C_21_H_18_O_12_ | [M+H]^+^ | 463.0871 | flavonoids | HQ |
| 118 | 58.67 | 3’-Hydroxydaidzin | C_15_H_10_O_5_ | [M+H]^+^ | 271.0595 | others | GG |
| 119 | 58.67 | Baicalin | C_21_H_18_O_11_ | [M+H]^+^ | 447.0921 | flavonoids | HQ |
| 120 | 58.67 | Baicalein | C_15_H_10_O_5_ | [M+H]^+^ | 271.0601 | flavonoids | HQ |
| 121 | 58.67 | Glychionide A | C_21_H_18_O_11_ | [M+H]^+^ | 447.0927 | flavonoids | HQ |
| 122 | 58.67 | Apigenin | C_15_H_10_O_5_ | [M+H]^+^ | 271.0600 | flavonoids | HQ |
| 123 | 58.67 | Apigenin7-O-Glucuronide | C_21_H_18_O_11_ | [M+H]^+^ | 447.0927 | flavonoids | HQ |
| 124 | 58.67 | Norwogonin | C_15_H_10_O_5_ | [M+H]^+^ | 271.0600 | flavonoids | HQ |
| 125 | 58.67 | Baicalein-6-O-Glucuronide | C_21_H_18_O_11_ | [M+H]^+^ | 447.0927 | flavonoids | HQ |
| 126 | 59.01 | Oroxylina-7-O-Glucuronidea | C_22_H_20_O_11_ | [M+H]^+^ | 461.1084 | flavonoids | HQ |
| 127 | 59.06 | Gancaonin | C_20_H_18_O_6_ | [M+H]^+^ | 355.1171 | flavonoids | GC |
| 128 | 59.64 | Dihydrooroxylin A | C_16_H_14_O_5_ | [M+H]^+^ | 287.0914 | flavonoids | HQ |
| 129 | 59.77 | Semilicoisoflavone A | C_20_H_16_O_6_ | [M+H]^+^ | 353.1019 | flavonoids | GC |
| 130 | 59.95 | Gancaonin A | C_21_H_20_O_5_ | [M+H]^+^ | 353.1383 | others | GC |

**Table S2.** Contents of 11 chemicals in fifteen batches of GQD.

| No. | Puerarin (mg/g) | Daidzin (mg/g) | Daidzein (mg/g) | Baicalin (mg/g) | Wogonoside (mg/g) | Baicalein (mg/g) | Wogonin (mg/g) | Berberine (mg/g) | Coptisine (mg/g) | Palmatine (mg/g) | Liquiritin (mg/g) |
| --- | --- | --- | --- | --- | --- | --- | --- | --- | --- | --- | --- |
| S1 | 10.938 | 1.711 | 0.226 | 6.880 | 2.121 | 0.014 | 0.012 | 1.062 | 0.423 | 0.756 | 1.822 |
| S2 | 8.561 | 1.205 | 0.117 | 4.709 | 2.114 | 0.020 | 0.021 | 1.154 | 0.421 | 0.748 | 1.191 |
| S3 | 8.544 | 1.208 | 0.127 | 5.714 | 2.114 | 0.011 | 0.022 | 0.742 | 0.224 | 0.495 | 1.826 |
| S4 | 9.779 | 1.789 | 0.244 | 5.358 | 2.112 | 0.013 | 0.022 | 0.776 | 0.243 | 0.625 | 1.788 |
| S5 | 8.751 | 1.298 | 0.229 | 6.010 | 2.194 | 0.012 | 0.013 | 1.014 | 0.382 | 0.741 | 1.954 |
| S6 | 9.646 | 1.321 | 0.202 | 6.885 | 2.191 | 0.018 | 0.022 | 0.821 | 0.399 | 0.634 | 1.598 |
| S7 | 9.142 | 1.318 | 0.201 | 6.882 | 2.121 | 0.017 | 0.016 | 0.962 | 0.301 | 0.644 | 1.952 |
| S8 | 9.224 | 1.048 | 0.141 | 5.725 | 2.188 | 0.014 | 0.014 | 0.861 | 0.301 | 0.632 | 1.653 |
| S9 | 8.695 | 1.293 | 0.189 | 5.342 | 2.117 | 0.014 | 0.013 | 0.630 | 0.265 | 0.497 | 1.831 |
| S10 | 9.698 | 1.755 | 0.242 | 6.909 | 2.198 | 0.021 | 0.015 | 0.631 | 0.271 | 0.455 | 1.795 |
| S11 | 9.282 | 1.281 | 0.141 | 6.909 | 2.199 | 0.022 | 0.015 | 0.532 | 0.337 | 0.466 | 1.188 |
| S12 | 8.031 | 1.111 | 0.135 | 6.881 | 2.116 | 0.017 | 0.016 | 0.716 | 0.295 | 0.498 | 1.666 |
| S13 | 8.803 | 1.378 | 0.194 | 6.367 | 2.186 | 0.013 | 0.013 | 0.767 | 0.265 | 0.497 | 1.785 |
| S14 | 8.580 | 1.273 | 0.140 | 6.895 | 2.192 | 0.018 | 0.022 | 0.954 | 0.320 | 0.743 | 1.657 |
| S15 | 8.681 | 1.138 | 0.125 | 7.369 | 2.197 | 0.022 | 0.014 | 0.791 | 0.307 | 0.633 | 1.713 |

**Table S3. The proteins of active compounds in GQD standard decoction.**

| Gene ID | Full Name | Symbol |
| --- | --- | --- |
| 132 | Adenosine kinase | ADK |
| 134 | Adenosine A1 receptor (by homology) | ADORA1 |
| 135 | Adenosine A2a receptor (by homology) | ADORA2A |
| 140 | Adenosine A3 receptor | ADORA3 |
| 151 | Alpha-2b adrenergic receptor | ADRA2B |
| 152 | Adrenoceptor alpha 2C | ADRA2C |
| 153 | Beta-1 adrenergic receptor | ADRB1 |
| 10327 | Aldehyde reductase (by homology) | AKR1A1 |
| 231 | Aldose reductase (by homology) | AKR1B1 |
| 1645 | Aldo-keto reductase family 1 member C1 (by homology) | AKR1C1 |
| 1646 | Aldo-keto reductase family 1 member C2 (by homology) | AKR1C2 |
| 8644 | Aldo-keto-reductase family 1 member C3 (by homology) | AKR1C3 |
| 1109 | Aldo-keto reductase family 1 member C4 (by homology) | AKR1C4 |
| 217 | Aldehyde dehydrogenase | ALDH2 |
| 239 | Arachidonate 12-lipoxygenase | ALOX12 |
| 246 | Arachidonate 15-lipoxygenase | ALOX15 |
| 9212 | Serine/threonine-protein kinase Aurora-B | AURKB |
| 554 | Vasopressin V2 receptor | AVPR2 |
| 23621 | Beta-secretase 1 | BACE1 |
| 759 | Carbonic anhydrase I | CA1 |
| 771 | Carbonic anhydrase XII | CA12 |
| 377677 | Carbonic anhydrase XIII | CA13 |
| 23632 | Carbonic anhydrase XIV | CA14 |
| 760 | Carbonic anhydrase II | CA2 |
| 761 | Carbonic anhydrase III | CA3 |
| 762 | Carbonic anhydrase IV | CA4 |
| 763 | Carbonic anhydrase VA | CA5A |
| 765 | Carbonic anhydrase VI | CA6 |
| 766 | Carbonic anhydrase VII | CA7 |
| 816 | Calmodulin dependent protein kinase II beta | CAMK2B |
| 873 | Carbonyl reductase [NADPH] 1 | CBR1 |
| 85417 | Cyclin-dependent kinase 1/cyclin B | CCNB3 |
| 1233 | C-C chemokine receptor type 4 | CCR4 |
| 8317 | Cell division cycle 7 | CDC7 |
| 1020 | Cyclin-dependent kinase 5 | CDK5 |
| 1128 | Muscarinic acetylcholine receptor M1 | CHRM1 |
| 1132 | Muscarinic acetylcholine receptor M4 | CHRM4 |
| 1139 | Neuronal acetylcholine receptor protein alpha-7 subunit | CHRNA7 |
| 1457 | Casein kinase II alpha | CSNK2A1 |
| 3577 | Interleukin-8 receptor A | CXCR1 |
| 1815 | Dopamine D4 receptor | DRD4 |
| 1973 | Eukaryotic initiation factor 4A-I | EIF4A1 |
| 2053 | Epoxide hydratase | EPHX2 |
| 2101 | Estrogen-related receptor alpha | ESRRA |
| 2103 | Estrogen-related receptor beta | ESRRB |
| 2339 | farnesyltransferase, CAAX box, alpha | FNTA |
| 2642 | Glucagon receptor | GCGR |
| 2739 | Glyoxalase I | GLO1 |
| 2859 | G-protein coupled receptor 35 | GPR35 |
| 2870 | G protein-coupled receptor kinase 6 | GRK6 |
| 3292 | Estradiol 17-beta-dehydrogenase 1 | HSD17B1 |
| 3294 | Estradiol 17-beta-dehydrogenase 2 | HSD17B2 |
| 3326 | Heat shock protein HSP 90-beta | HSP90AB1 |
| 3357 | Serotonin 2b (5-HT2b) receptor | HTR2B |
| 3358 | Serotonin 2c (5-HT2c) receptor | HTR2C |
| 3778 | potassium large conductance calcium-activated channel, subfamily M, alpha member 1 | KCNMA1 |
| 390245 | Lysine-specific demethylase 4D-like | KDM4E |
| 4129 | Monoamine oxidase B | MAOB |
| 8972 | Maltase-glucoamylase | MGAM |
| 4321 | Matrix metalloproteinase 12 | MMP12 |
| 4322 | Matrix metalloproteinase 13 | MMP13 |
| 10499 | Nuclear receptor coactivator 2 | NCOA2 |
| 4751 | Serine/threonine-protein kinase NEK2 | NEK2 |
| 10783 | Serine/threonine-protein kinase NEK6 | NEK6 |
| 50507 | NADPH oxidase 4 | NOX4 |
| 9891 | NUAK family SNF1-like kinase 1 | NUAK1 |
| 4985 | Delta opioid receptor | OPRD1 |
| 5144 | Phosphodiesterase 4D | PDE4D |
| 8654 | Phosphodiesterase 5A | PDE5A |
| 5170 | 3-phosphoinositide dependent protein kinase-1 | PDPK1 |
| 5209 | 6-phosphofructo-2-kinase/fructose-2,6-bisphosphatase 3 | PFKFB3 |
| 5292 | Serine/threonine-protein kinase PIM1 | PIM1 |
| 5585 | Protein kinase N1 | PKN1 |
| 5319 | Phospholipase A2 group 1B | PLA2G1B |
| 5644 | Protease, serine, 1 (trypsin 1) | PRSS1 |
| 5770 | Protein-tyrosine phosphatase 1B | PTPN1 |
| 5836 | Liver glycogen phosphorylase | PYGL |
| 5914 | Retinoic acid receptor alpha | RARA |
| 10055 | SUMO1 activating enzyme subunit 1 | SAE1 |
| 6462 | Testis-specific androgen-binding protein | SHBG |
| 10280 | Sigma opioid receptor | SIGMAR1 |
| 116085 | Solute carrier family 22 member 12 | SLC22A12 |
| 9153 | Sodium/nucleoside cotransporter 2 | SLC28A2 |
| 64078 | Solute carrier family 28 member 3 | SLC28A3 |
| 6524 | Sodium/glucose cotransporter 2 | SLC5A2 |
| 6527 | Low affinity sodium-glucose cotransporter | SLC5A4 |
| 6715 | Steroid 5-alpha-reductase 1 | SRD5A1 |
| 412 | steroid sulfatase | STS |
| 259290 | Taste receptor type 2 member 31 | TAS2R31 |
| 6916 | Thromboxane-A synthase | TBXAS1 |
| 8658 | Tankyrase-1 | TNKS |
| 80351 | Tankyrase-2 | TNKS2 |
| 7155 | DNA topoisomerase II | TOP2B |
| 7514 | Exportin-1 | XPO1 |

**Table S4.** The 74 potential proteins of GQD standard decoction for the treatment of CID.

| Gene ID | Full Name | Symbol |
| --- | --- | --- |
| 5243 | P-glycoprotein 1 | ABCB1 |
| 4915 | Neurotrophic tyrosine kinase receptor type 2 | NTRK2 |
| 4363 | Multidrug resistance-associated protein 1 | ABCC1 |
| 4233 | Hepatocyte growth factor receptor | MET |
| 9429 | ATP-binding cassette sub-family G member 2 | ABCG2 |
| 25 | Tyrosine-protein kinase ABL | ABL1 |
| 7015 | Telomerase reverse transcriptase | TERT |
| 43 | Acetylcholinesterase | ACHE |
| 154 | Adrenergic receptor beta | ADRB2 |
| 207 | Serine/threonine-protein kinase AKT | AKT1 |
| 5347 | Serine/threonine-protein kinase PLK1 | PLK1 |
| 238 | ALK tyrosine kinase receptor | ALK |
| 240 | Arachidonate 5-lipoxygenase | ALOX5 |
| 351 | Beta amyloid A4 protein | APP |
| 367 | Androgen receptor | AR |
| 6790 | Serine/threonine-protein kinase Aurora-A | AURKA |
| 5465 | Peroxisome proliferator-activated receptor alpha | PPARA |
| 558 | Tyrosine-protein kinase receptor UFO | AXL |
| 7299 | Tyrosinase | TYR |
| 590 | Butyrylcholinesterase | BCHE |
| 768 | Carbonic anhydrase IX | CA9 |
| 998 | Cell division control protein 42 homolog | CDC42 |
| 983 | Cyclin-dependent kinase 1 | CDK1 |
| 1021 | Cyclin-dependent kinase 6 | CDK6 |
| 3577 | Interleukin-8 receptor A | CXCR1 |
| 1588 | Cytochrome P450 19A1 | CYP19A1 |
| 1543 | Cytochrome P450 1A1 | CYP1A1 |
| 1544 | Cytochrome P450 1A2 | CYP1A2 |
| 1545 | Cytochrome P450 1B1 | CYP1B1 |
| 1565 | Cytochrome P450 2D6 | CYP2D6 |
| 1612 | Death-associated protein kinase 1 | DAPK1 |
| 1803 | Dipeptidyl peptidase 4 | DPP4 |
| 1956 | Epidermal growth factor receptor erbB1 | EGFR |
| 2099 | Estrogen receptor alpha | ESR1 |
| 2100 | Estrogen receptor beta | ESR2 |
| 7430 | Ezrin | EZR |
| 2147 | Thrombin | F2 |
| 2152 | Coagulation factor VII/tissue factor | F3 |
| 2322 | Tyrosine-protein kinase receptor FLT3 | FLT3 |
| 2932 | Glycogen synthase kinase-3 beta | GSK3B |
| 3320 | Heat shock protein HSP 90-alpha | HSP90AA1 |
| 7184 | Heat shock protein 90 beta family member 1 | HSP90B1 |
| 3356 | Serotonin 2a (5-HT2a) receptor | HTR2A |
| 3480 | Insulin-like growth factor I receptor | IGF1R |
| 3551 | Inhibitor of nuclear factor kappa B kinase beta subunit | IKBKB |
| 3558 | Interleukin-2 | IL2 |
| 3791 | Vascular endothelial growth factor receptor 2 | KDR |
| 3815 | Stem cell growth factor receptor | KIT |
| 4128 | Monoamine oxidase A | MAOA |
| 4170 | Induced myeloid leukemia cell differentiation protein Mcl-1 | MCL1 |
| 4282 | Macrophage migration inhibitory factor | MIF |
| 4312 | Matrix metalloproteinase 1 | MMP1 |
| 4313 | Matrix metalloproteinase 2 | MMP2 |
| 4314 | Matrix metalloproteinase 3 | MMP3 |
| 4318 | Matrix metalloproteinase 9 | MMP9 |
| 4353 | Myeloperoxidase | MPO |
| 4790 | Nuclear factor NF-kappa-B p105 subunit | NFKB1 |
| 4843 | Nitric oxide synthase, inducible | NOS2 |
| 4953 | Ornithine decarboxylase | ODC1 |
| 4988 | Mu opioid receptor | OPRM1 |
| 5295 | PI3-kinase p85-alpha subunit | PIK3R1 |
| 5551 | Perforin-1 | PRF1 |
| 5742 | Cyclooxygenase-1 | PTGS1 |
| 5743 | Cyclooxygenase-2 | PTGS2 |
| 5747 | Focal adhesion kinase 1 | PTK2 |
| 2185 | Protein tyrosine kinase 2 beta | PTK2B |
| 5879 | Ras-related C3 botulinum toxin substrate 1 | RAC1 |
| 5894 | Serine/threonine-protein kinase RAF | RAF1 |
| 2030 | Solute carrier family 29 member 1 (Augustine blood group) | SLC29A1 |
| 6523 | Solute carrier family 5 member 1 | SLC5A1 |
| 6714 | Tyrosine-protein kinase SRC | SRC |
| 7124 | TNF-alpha | TNF |
| 7494 | X-box-binding protein 1 | XBP1 |
| 7498 | Xanthine dehydrogenase | XDH |

**Table S5. KEGG enrichment analysis results.**

| ID | Description | P Value | Count |
| --- | --- | --- | --- |
| hsa05215 | Prostate cancer | 2.15E-12 | 13 |
| hsa05417 | Lipid and atherosclerosis | 3.98E-12 | 17 |
| hsa04151 | PI3K-Akt signaling pathway | 1.91E-11 | 20 |
| hsa05205 | Proteoglycans in cancer | 2.29E-11 | 16 |
| hsa05207 | Chemical carcinogenesis - receptor activation | 3.81E-11 | 16 |
| hsa01522 | Endocrine resistance | 4.87E-11 | 12 |
| hsa05418 | Fluid shear stress and atherosclerosis | 2.24E-10 | 13 |
| hsa04014 | Ras signaling pathway | 1.49E-09 | 15 |
| hsa01521 | EGFR tyrosine kinase inhibitor resistance | 1.62E-09 | 10 |
| hsa04370 | VEGF signaling pathway | 2.05E-09 | 9 |
| hsa05135 | Yersinia infection | 2.53E-09 | 12 |
| hsa04915 | Estrogen signaling pathway | 2.75E-09 | 12 |
| hsa05208 | Chemical carcinogenesis - reactive oxygen species | 8.23E-09 | 14 |
| hsa04657 | IL-17 signaling pathway | 9.14E-09 | 10 |
| hsa05163 | Human cytomegalovirus infection | 9.24E-09 | 14 |
| hsa05212 | Pancreatic cancer | 2.05E-08 | 9 |
| hsa05219 | Bladder cancer | 6.28E-08 | 7 |
| hsa05206 | MicroRNAs in cancer | 7.55E-08 | 15 |
| hsa04722 | Neurotrophin signaling pathway | 9.00E-08 | 10 |
| hsa04062 | Chemokine signaling pathway | 1.14E-07 | 12 |
| hsa05221 | Acute myeloid leukemia | 1.24E-07 | 8 |
| hsa04917 | Prolactin signaling pathway | 1.76E-07 | 8 |
| hsa05120 | Epithelial cell signaling in Helicobacter pylori infection | 1.76E-07 | 8 |
| hsa04510 | Focal adhesion | 1.88E-07 | 12 |
| hsa04926 | Relaxin signaling pathway | 1.94E-07 | 10 |
| hsa04010 | MAPK signaling pathway | 2.67E-07 | 14 |
| hsa04625 | C-type lectin receptor signaling pathway | 3.24E-07 | 9 |
| hsa04660 | T cell receptor signaling pathway | 3.24E-07 | 9 |
| hsa05224 | Breast cancer | 6.58E-07 | 10 |
| hsa04012 | ErbB signaling pathway | 8.04E-07 | 8 |
| hsa04932 | Non-alcoholic fatty liver disease | 1.07E-06 | 10 |
| hsa05160 | Hepatitis C | 1.21E-06 | 10 |
| hsa05222 | Small cell lung cancer | 1.48E-06 | 8 |
| hsa04015 | Rap1 signaling pathway | 2.35E-06 | 11 |
| hsa05230 | Central carbon metabolism in cancer | 2.72E-06 | 7 |
| hsa04933 | AGE-RAGE signaling pathway in diabetic complications | 2.79E-06 | 8 |
| hsa04914 | Progesterone-mediated oocyte maturation | 3.24E-06 | 8 |
| hsa05218 | Melanoma | 3.30E-06 | 7 |
| hsa05223 | Non-small cell lung cancer | 3.30E-06 | 7 |
| hsa05220 | Chronic myeloid leukemia | 4.75E-06 | 7 |
| hsa04913 | Ovarian steroidogenesis | 5.64E-06 | 6 |
| hsa01523 | Antifolate resistance | 6.15E-06 | 5 |
| hsa05165 | Human papillomavirus infection | 6.47E-06 | 13 |
| hsa04668 | TNF signaling pathway | 6.55E-06 | 8 |
| hsa04670 | Leukocyte transendothelial migration | 7.47E-06 | 8 |
| hsa04662 | B cell receptor signaling pathway | 7.92E-06 | 7 |
| hsa04726 | Serotonergic synapse | 7.97E-06 | 8 |
| hsa05167 | Kaposi sarcoma-associated herpesvirus infection | 8.14E-06 | 10 |
| hsa05131 | Shigellosis | 1.12E-05 | 11 |
| hsa05161 | Hepatitis B | 1.32E-05 | 9 |
| hsa05235 | PD-L1 expression and PD-1 checkpoint pathway in cancer | 1.37E-05 | 7 |
| hsa05225 | Hepatocellular carcinoma | 1.76E-05 | 9 |
| hsa05170 | Human immunodeficiency virus 1 infection | 1.77E-05 | 10 |
| hsa04210 | Apoptosis | 2.73E-05 | 8 |
| hsa04664 | Fc epsilon RI signaling pathway | 3.04E-05 | 6 |
| hsa05211 | Renal cell carcinoma | 3.30E-05 | 6 |
| hsa04360 | Axon guidance | 3.33E-05 | 9 |
| hsa05142 | Chagas disease | 3.34E-05 | 7 |
| hsa04520 | Adherens junction | 3.89E-05 | 6 |
| hsa04931 | Insulin resistance | 4.83E-05 | 7 |
| hsa04072 | Phospholipase D signaling pathway | 5.01E-05 | 8 |
| hsa05226 | Gastric cancer | 5.26E-05 | 8 |
| hsa05214 | Glioma | 5.32E-05 | 6 |
| hsa05100 | Bacterial invasion of epithelial cells | 6.17E-05 | 6 |
| hsa05130 | Pathogenic Escherichia coli infection | 6.20E-05 | 9 |
| hsa04071 | Sphingolipid signaling pathway | 8.96E-05 | 7 |
| hsa05210 | Colorectal cancer | 0.000115 | 6 |
| hsa04810 | Regulation of actin cytoskeleton | 0.000135 | 9 |
| hsa04923 | Regulation of lipolysis in adipocytes | 0.000137 | 5 |
| hsa05213 | Endometrial cancer | 0.000162 | 5 |
| hsa04068 | FoxO signaling pathway | 0.000164 | 7 |
| hsa04912 | GnRH signaling pathway | 0.000177 | 6 |
| hsa05171 | Coronavirus disease - COVID-19 | 0.000216 | 9 |
| hsa05162 | Measles | 0.000237 | 7 |
| hsa05202 | Transcriptional misregulation in cancer | 0.000307 | 8 |
| hsa04620 | Toll-like receptor signaling pathway | 0.000326 | 6 |
| hsa05132 | Salmonella infection | 0.000365 | 9 |
| hsa04920 | Adipocytokine signaling pathway | 0.000369 | 5 |
| hsa04066 | HIF-1 signaling pathway | 0.00042 | 6 |
| hsa05415 | Diabetic cardiomyopathy | 0.000446 | 8 |
| hsa04150 | mTOR signaling pathway | 0.000478 | 7 |
| hsa05145 | Toxoplasmosis | 0.000486 | 6 |
| hsa00380 | Tryptophan metabolism | 0.000522 | 4 |
| hsa05010 | Alzheimer disease | 0.000584 | 11 |
| hsa04919 | Thyroid hormone signaling pathway | 0.000733 | 6 |
| hsa05164 | Influenza A | 0.000827 | 7 |
| hsa04380 | Osteoclast differentiation | 0.000984 | 6 |
| hsa04650 | Natural killer cell mediated cytotoxicity | 0.001111 | 6 |
| hsa04540 | Gap junction | 0.001127 | 5 |
| hsa04020 | Calcium signaling pathway | 0.001336 | 8 |
| hsa04613 | Neutrophil extracellular trap formation | 0.001529 | 7 |
| hsa04936 | Alcoholic liver disease | 0.001682 | 6 |
| hsa04666 | Fc gamma R-mediated phagocytosis | 0.001742 | 5 |
| hsa05231 | Choline metabolism in cancer | 0.001823 | 5 |
| hsa00140 | Steroid hormone biosynthesis | 0.002139 | 4 |
| hsa05169 | Epstein-Barr virus infection | 0.00217 | 7 |
| hsa05146 | Amoebiasis | 0.002175 | 5 |
| hsa05203 | Viral carcinogenesis | 0.002295 | 7 |
| hsa04929 | GnRH secretion | 0.002552 | 4 |
| hsa04218 | Cellular senescence | 0.002706 | 6 |
| hsa04630 | JAK-STAT signaling pathway | 0.003265 | 6 |
| hsa05204 | Chemical carcinogenesis - DNA adducts | 0.003357 | 4 |
| hsa04024 | cAMP signaling pathway | 0.00359 | 7 |
| hsa05166 | Human T-cell leukemia virus 1 infection | 0.00368 | 7 |
| hsa00350 | Tyrosine metabolism | 0.004035 | 3 |
| hsa04935 | Growth hormone synthesis, secretion and action | 0.004239 | 5 |
| hsa05330 | Allograft rejection | 0.004706 | 3 |
| hsa05140 | Leishmaniasis | 0.004979 | 4 |
| hsa04611 | Platelet activation | 0.005048 | 5 |
| hsa00980 | Metabolism of xenobiotics by cytochrome P450 | 0.005213 | 4 |
| hsa04110 | Cell cycle | 0.0054 | 5 |
| hsa05152 | Tuberculosis | 0.005464 | 6 |
| hsa05332 | Graft-versus-host disease | 0.006242 | 3 |
| hsa04114 | Oocyte meiosis | 0.006357 | 5 |
| hsa04940 | Type I diabetes mellitus | 0.006668 | 3 |
| hsa02010 | ABC transporters | 0.007569 | 3 |
| hsa04910 | Insulin signaling pathway | 0.007656 | 5 |
| hsa04930 | Type II diabetes mellitus | 0.008045 | 3 |
| hsa04211 | Longevity regulating pathway | 0.008288 | 4 |
| hsa04973 | Carbohydrate digestion and absorption | 0.008539 | 3 |
| hsa04140 | Autophagy - animal | 0.008618 | 5 |
| hsa00360 | Phenylalanine metabolism | 0.008835 | 2 |
| hsa04550 | Signaling pathways regulating pluripotency of stem cells | 0.00913 | 5 |
| hsa00330 | Arginine and proline metabolism | 0.01069 | 3 |
| hsa04916 | Melanogenesis | 0.012788 | 4 |
| hsa04064 | NF-kappa B signaling pathway | 0.014115 | 4 |
| hsa04659 | Th17 cell differentiation | 0.016017 | 4 |
| hsa05416 | Viral myocarditis | 0.016587 | 3 |
| hsa00590 | Arachidonic acid metabolism | 0.017334 | 3 |
| hsa04213 | Longevity regulating pathway - multiple species | 0.0181 | 3 |
| hsa05321 | Inflammatory bowel disease | 0.02051 | 3 |
| hsa04622 | RIG-I-like receptor signaling pathway | 0.024901 | 3 |
| hsa05022 | Pathways of neurodegeneration - multiple diseases | 0.026212 | 9 |
| hsa00982 | Drug metabolism - cytochrome P450 | 0.026788 | 3 |
| hsa05133 | Pertussis | 0.030787 | 3 |
