# Supplementary material for: Gegen Qinlian standard decoction alleviated irinotecan-induced diarrhea via PI3K/AKT/NF-κB axis by network pharmacology prediction and experimental validation combination
Source: Chin Med. 2023 Apr 27;18:46. doi: 10.1186/s13020-023-00747-3 (PMC10134581; doi:10.1186/s13020-023-00747-3)
Supplement: Supplementary file 2 — Additional file 2: Figure S1. Mass spectrum chromatograms of reference standards. [file 13020_2023_747_MOESM2_ESM.docx]

**Gegen Qinlian standard decoction alleviated** **irinotecan-induced diarrhea via PI3K/AKT/NF-κB axis by network pharmacology prediction and experimental validation combination**

**Supplementary Figure**

**Fig. S1.** Mass spectrum chromatograms of reference standards.


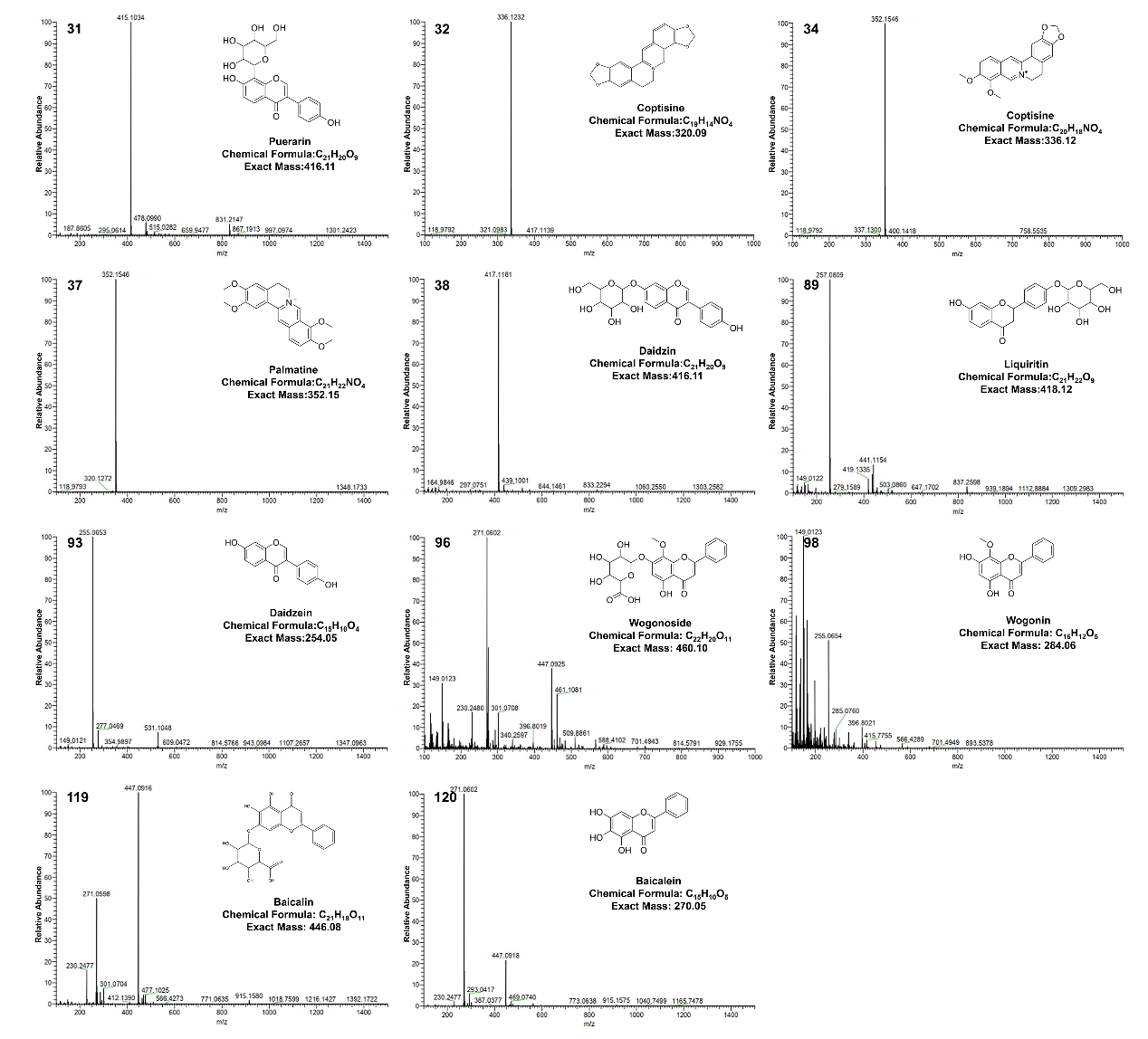


**Fig. S1.** Mass spectrum chromatograms of reference standards. MS spectra of representative compound, including Puerarin, Coptisine, Berberine, Palmatine, Daidzin, Liquiritin, Daidzein, Wogonoside, Wogonin, Baicalin, Baicalein.
